# Fly Photoreceptors Encode Phase Congruency

**DOI:** 10.1371/journal.pone.0157993

**Published:** 2016-06-23

**Authors:** Uwe Friederich, Stephen A. Billings, Roger C. Hardie, Mikko Juusola, Daniel Coca

**Affiliations:** 1 Department of Automatic Control & Systems Engineering, the University of Sheffield, Mappin Street, Sheffield, S1 3JD, United Kingdom; 2 Department of Physiology, Development and Neuroscience, University of Cambridge, Downing Street, Cambridge, CB2 3DY, United Kingdom; 3 Department of Biomedical Science, the University of Sheffield, Western Bank, Sheffield, S10 2TN, United Kingdom; University College London, UNITED KINGDOM

## Abstract

More than five decades ago it was postulated that sensory neurons detect and selectively enhance behaviourally relevant features of natural signals. Although we now know that sensory neurons are tuned to efficiently encode natural stimuli, until now it was not clear what statistical features of the stimuli they encode and how. Here we reverse-engineer the neural code of *Drosophila* photoreceptors and show for the first time that photoreceptors exploit nonlinear dynamics to selectively enhance and encode phase-related features of temporal stimuli, such as local phase congruency, which are invariant to changes in illumination and contrast. We demonstrate that to mitigate for the inherent sensitivity to noise of the local phase congruency measure, the nonlinear coding mechanisms of the fly photoreceptors are tuned to suppress random phase signals, which explains why photoreceptor responses to naturalistic stimuli are significantly different from their responses to white noise stimuli.

## Introduction

In his seminal papers [1][2] Horace Barlow postulated that sensory pathways are tuned to detect, enhance, and efficiently encode the stimuli that are important for survival. However, as Barlow pointed out [1], the signal transformations performed by sensory neurons are difficult to characterize using ordinary physiological investigations. In particular, the responses of sensory neurons contain components from linear and nonlinear transductions that are difficult to separate [3].

Although it has been known for some time that sensory neurons, whether auditory [4][5][6], olfactory [7] or visual [8][9][10], respond nonlinearly when driven by stimuli that have ‘naturalistic’ properties, the nonlinear relationship between the statistical properties of the stimuli and the neuron responses—or in other words, the computations performed by these neurons [11]—have not been fully characterized.

In the visual system, the detection of the boundary or edges of objects is crucial for object segregation, categorization and recognition as well as for motion detection [12]. In this context, the temporal structure of the retinal images is very important [12] since moving spatial edges generated by self-motion or by moving objects produce temporal edges at the photoreceptor level. Therefore, selectively enhancing the salience of these temporal features should facilitate downstream processing of the spatio-temporal visual stimuli for motion detection. As edges correspond to points of local maximum phase alignment of the constituent Fourier components, local phase congruency, which is invariant to illumination and contrast, is an accurate measure of edge saliency that can be encoded by the photoreceptors [13][14][15].

There are many clues, which suggest photoreceptors are tuned to distinguish and selectively process the temporal phase correlations present in light stimuli. These phase correlations are biologically relevant [4][16][17].

It is well known, for example, that photoreceptor responses to naturalistic stimuli are highly nonlinear [8] whereas Gaussian white noise stimuli tend to linearize the response [18].

Naturalistic stimuli exhibit local and global phase correlations caused by the edges, contours and textures present in the natural scene [12,19], which can be described by higher-order statistics [20–23] and can only be encoded by applying nonlinear transformations to the stimuli [24].

In contrast, Gaussian white noise signals exhibit no phase correlations and are completely characterised by first- and second-order statistics. Because white noise stimuli lack higher-order correlations present in natural scenes, linear encoding ensures that photoreceptors do not generate spurious higher-order correlations that the fly brain would use to distinguish environmental features.

Experimental and modelling studies investigating the role of photoreceptors in the detection of moving point objects [25–27] provide further evidence that photoreceptors contribute to the enhancement of image features that are essential for object recognition. The photoreceptors of male houseflies, for example, exhibit surprisingly large responses to moving targets [28] which cannot be explained by linear models derived from photoreceptor responses to white noise stimuli. This highlights the need to use naturalistic-like stimuli with a higher-order statistical structure, in order to characterize the nonlinear encoding mechanisms of photoreceptors [29].

More recent work [16] has shown that temporal processing performed by photoreceptors of male hoverflies (*Eristalis tenax)* enhances not only the moving target but also relatively static features present in the background, which are important for navigational purposes. Although this work highlights the importance of nonlinear processing in target detection, the relationship between the image features and the nonlinear component of the response is not elucidated.

The current paper provides a quantitative characterization of the relationship between the statistical properties of environmental stimuli and fly photoreceptor responses. The study is based on a nonlinear dynamical model that predicts accurately the responses of individual fly photoreceptors to white noise and naturalistic stimuli, for the entire environmental range of light intensities.

Higher-order frequency response functions analytically derived from the model equations are then used to characterize the nonlinear transformations that enable *Drosophila* photoreceptors to encode measures of dependence between phase angles of different frequency components of the temporal stimuli, which are invariant to contrast and illumination, such as local phase congruency. We argue that these phase-related measures, which are encoded nonlinearly, may facilitate the identification of behaviourally important features in the natural scenes.

By carrying out a comparative signal-to-noise analysis of the linear and nonlinear components of the response, we show that photoreceptors are tuned to selectively improve the Signal-to-Noise Ratio (SNR) of the nonlinear component of the photoreceptor response, which encodes the local phase congruency measure. This explains why the photoreceptor responses to naturalistic stimuli are significantly different from their responses to white noise stimuli.

To validate the results, we carried out electrophysiological experiments using temporal stimuli that allow us to separate the nonlinear component of the photoreceptor responses, which encode local phase congruency, directly from the measured responses. It is shown that our model correctly predicts the measured nonlinear component of the photoreceptor response, elicited by local and non-local phase correlations introduced deliberately in the synthetic stimuli.

A similar analysis carried out using recordings from blind *hdc*^JK910^ mutant flies, indicates that the nonlinear transformations underlying the detection of phase correlations in the temporal light stimuli are performed by phototransduction alone, and do not require synaptic interactions between neighbouring neurons.

The results could have important implications beyond fly photoreceptors. A similar nonlinear encoding strategy may well be implemented in the mammalian retina or in other types of sensory neurons.

## Materials and Methods

### Electrophysiology

Flies were prepared for intracellular *in vivo* recordings from blue-green sensitive R1-R6 photoreceptors according to previously described methods [9,30]. To present the temporal stimulus pattern at different light levels, we designed a computer controlled point light source with two converging light paths (S1 Fig). In each path, LED drivers with light feedback (Cairn Research, model OptoLED) assured a linear relation between the light pattern, stored on a computer and the output of high power LED’s (Seoul, model Z-Power LED P4, white, 240 lm). Neutral density filters (Kodak Wratten, ND gel filters) were used to generate 5 distinct light intensity levels, from *L*_0_ (bright) to *L*_-4_ (very dark). Only one path was active at a time. This allowed modifying the filter setting of the inactive path in real time. Step changes of light intensity were thus achieved by switching between the two paths implementing different filter settings. Both LED drivers were carefully calibrated to produce exactly the same light output for a given reference signal and filter setting.

The amplified temporal light stimuli (inputs) and voltage responses (outputs), sampled at 2 kHz, were low-pass filtered by analogue low-pass elliptic filters (KEMO Limited, model VBF/23) with a 500 Hz cut-off before being used to drive the LEDs or to perform A/D conversion respectively. The measured output of the LEDs was considered to be the input to the photoreceptor. A/D and D/A conversions (12bit resolution) were performed using National Instruments A/D and D/A boards (PCI M-IO 16E4 and PCI 6713). Custom written software was used to interface the NI boards with MATLAB (Mathworks, R7.14). For modelling and analysis purposes the data was down‐sampled to 400 Hz, which provided sufficient bandwidth to capture in full the photoreceptor dynamics.

### Stimuli

To characterize in full the nonlinear photoreceptor dynamics, we used naturalistic input stimuli that resemble the light fluctuations these cells are subjected to in a fly’s natural habitat. Stimulus sequences with an average power spectrum *S*(*f*) = 1/*f* typical for natural images were selected from the van Hateren stimulus collection [29].

**Fig 1:**
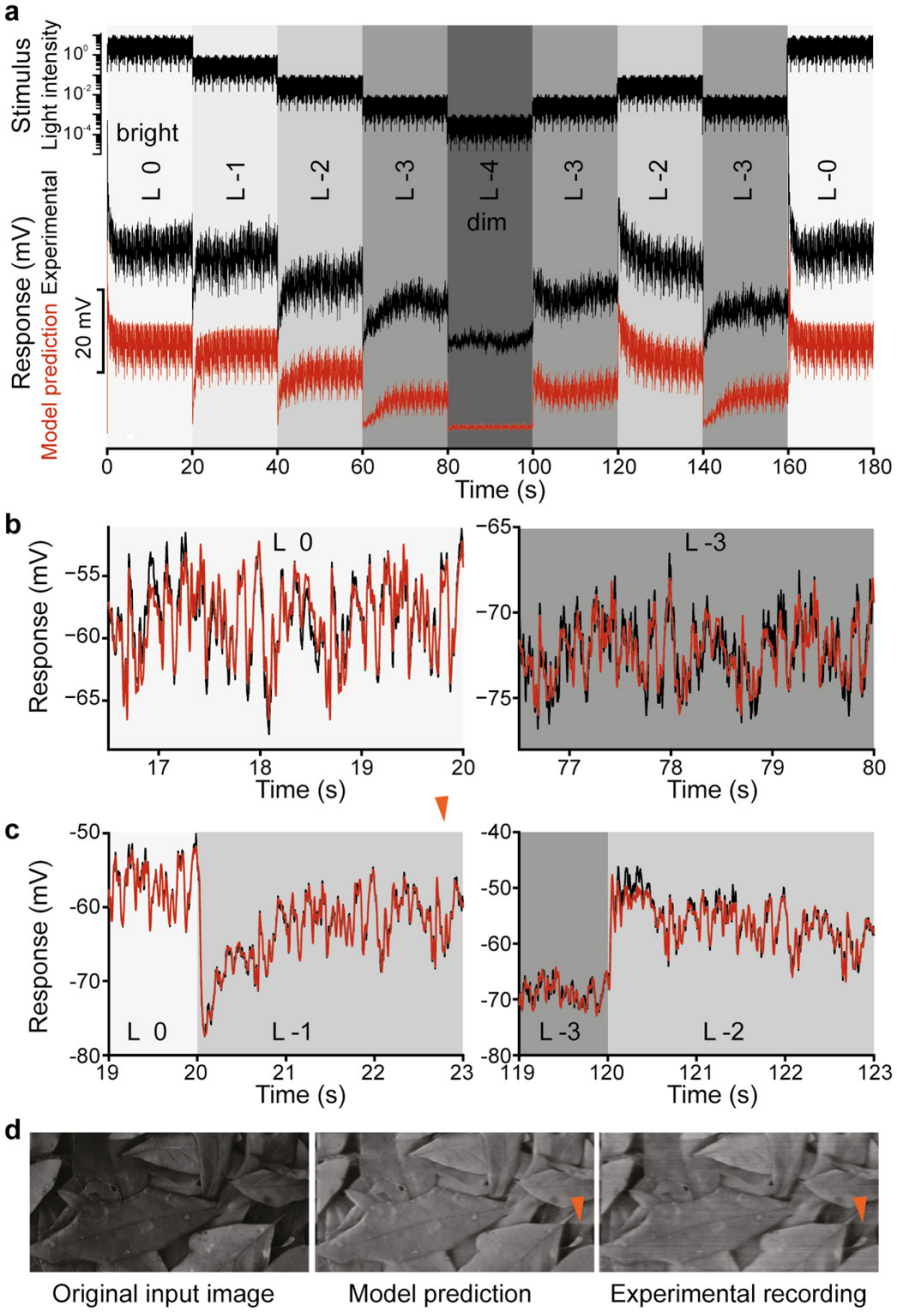
Evaluation of the adaptive photoreceptor model. **a**, Multi-level naturalistic stimulus sequence (top); *in vivo* experimental recordings from a R1-R6 *Drosophila* photoreceptor (black) and the photoreceptor model predictions (red) shown with an offset of -25 mV for comparison. **b**, Short segments of stationary data: experimental (black) and model predicted (red) for two mean intensity levels. **c**, Model predictions (red) superimposed on experimental response measurements (black) during transient regimes illustrate the performance of the gain control model. **d**, Images reconstructed from the model predicted response time-series (middle) and the *in vivo* measured response time-series (left) corresponding to a temporal stimulus sequence generated by scanning line-by-line the image on the left. The artifacts in the reconstructed images (indicated by a red arrow) reflect photoreceptor adaptation to sharp bright-to-dim changes of intensity values of pixels along the scanned line in the image.

To derive the adaptation rules over the full operating range, we concatenated stimulus sequences with different mean intensity levels as shown in Fig 1a, which allowed us to characterize the transient photoreceptor responses to step changes of mean light intensity.

To illustrate further the validity of the model, we generated a synthetic temporal stimulus by scanning line-by-line an image (Fig 1d). The stimulus was used to excite both the final photoreceptor model as well as measure *in vivo*, fly photoreceptor responses. The model simulation and the electrophysiological recordings were subsequently used to reassemble the corresponding processed images for comparison.

The synthetic stimulus sequence mimics what a photoreceptor would experience as a fly moves through a natural scene, containing shadows and sunlit areas. As light intensities in such a scene can vary up to 10,000-fold, we modified our relative illumination range accordingly in 5 distinct logarithmic levels (*L*_0_ = bright to *L*_-4_ = very dark).

### Model Development

The photoreceptor model was derived directly from electrophysiological recordings using the nonlinear system identification methodology [31,32] based on the NARMAX (Nonlinear AutoRegressive Moving Average with eXogenous inputs) model, which is applicable to a wide class of nonlinear dynamical systems. The Wiener (Linear-Nonlinear) and Hammerstein (Nonlinear-Linear) models, that are routinely used in neuroscience, can be viewed as special types of NARMAX models. Notable advantages of NARMAX methodology include the fact that it does not require knowledge of the model structure and that the noise is modelled explicitly to ensure unbiased estimates of model parameters, even if the noise is not additive or white.

The photoreceptor model consists of a polynomial discrete-time NARMAX model with variable input gain complemented by a dynamic gain control model with three adaptation time-scales (S1 File and S5 Fig). The nonlinear model with an appropriately adjusted gain fully characterizes the photoreceptor dynamics for stimuli with constant mean intensity for the entire operating range. The gain control model captures the dynamic relationship between the stimulus and the gain of the photoreceptor while it adapts to changes in mean light intensity. The three control loops reflect the dynamics of different biochemical mechanisms of light adaptation (S1 File) which we characterized in our detailed biophysical model of the photoreceptor [33].

### Photoreceptor Response Decomposition using the Generalized Frequency Response Functions

**Fig 2:**
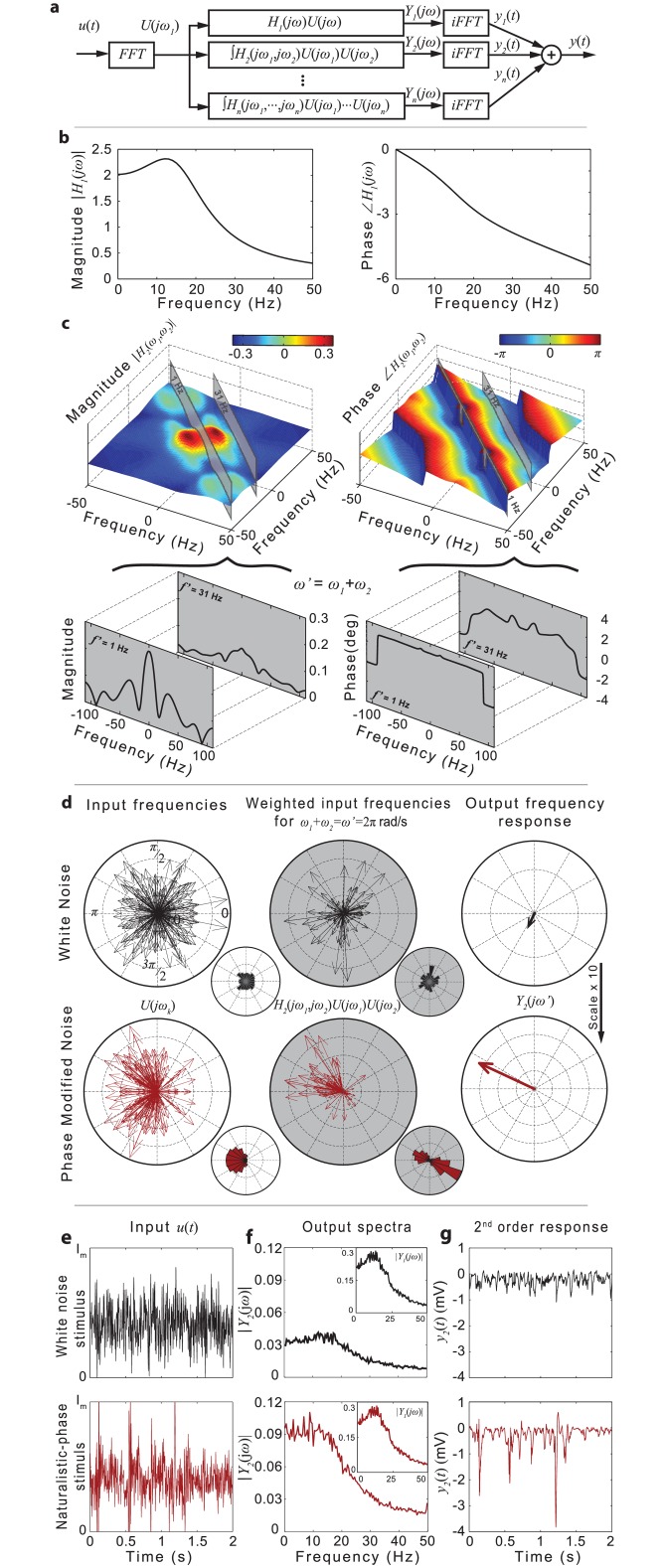
Output frequency response decomposition elucidates differences in photoreceptor coding white noise and naturalistic stimuli. **a**, Block diagram illustrating the output frequency response decomposition approach. **b**,**c**, Plots of the magnitude and phase for the first- and second-order frequency response functions. Slices through the second-order magnitude and phase functions are taken along the integration lines given by *f*_1_+ *f*_2_ = 1Hz and *f*_1_+ *f*_2_ = 31Hz. **d**, Polar plots and distributions of the Fourier components *U*(*jω*) of the white noise (black) and naturalistic phase (red) stimuli before and after weighting by *H*_2_(*jω*_1_, *jω*_2_) for pairs of input frequencies (*ω*_1_, *ω*_2_) satisfying *ω*_1_+*ω*_2_ = *ω’* = 2π rad/s. The total output frequency component *Y*_2_(*jω’*) for white noise (black) and naturalistic (red) stimuli obtained by integrating *H*_2_(*jω*_1_, *jω*_2_) *U*(*jω*_1_) *U*(*jω*_2_) along the line *ω*_1_+*ω*_2_ = *ω’* = 2π. **e**, White noise (top, black line) and naturalistic- phase (bottom, red line) stimuli. **f**, Output magnitude spectra |*Y*_1_(*jω*)| and |*Y*_2_(*jω*)| of the first- and second-order responses, *y*_1_(*t*) and *y*_2_(*t*) to white-noise (top, black line) and naturalistic-phase (bottom, red line) stimuli. **g**, The second-order component *y*_2_(*t*) of the model response *y*(*t*) for white noise (top, black) and naturalistic-phase (bottom, red line) stimuli.

Compared with previous models of fly photoreceptors [8,34], the current model not only predicts the photoreceptor responses over the entire environmental range of stimuli but also models explicitly the relationship between the stimulus intensity and the dynamic gain of the nonlinear NARMAX filter. The model can also be used to characterize the nonlinear transformations performed by photoreceptors by deriving analytically the generalized frequency response functions [35–37] (GFRFs) (Fig 2a–2c) of the system. GFRF’s, which are extensions to the classical linear frequency response function, characterize the linear and nonlinear relationships between the photoreceptor’s input and output spectral components.

The approach is similar to that adopted in their pioneering work by Victor and Shapley [3], investigating the receptive field mechanisms of retinal ganglion cells, and by French *et al* [34] and Asyali and Juusola [38], in their study of *Drosophila* photoreceptor. Victor and Shapley [3] applied stimuli composed of sums of sinusoids, to estimate, directly from data, the first and second order kernels of a Wiener series expansion of the response. In the case of the fruit fly, French *et al* [34] and Asyali and Juusola [38] used steps and white noise sequences, respectively, to elicit photoreceptor responses and fit first and second order Volterra kernels.

In contrast, here we use a dynamical model to derive analytically the GFRFs i.e. the Fourier Transforms of the kernels of the Volterra series associated with the nonlinear system (S1 File).

The main novelty of our analysis is that we use the GFRFs to compute spectral and temporal decompositions of the response, which allow us to provide an analytical interpretation of the role played by the nonlinear transductions at photoreceptor level rather than in the neurons downstream of photoreceptors. This provides a unique insight into the nonlinear encoding algorithms implemented by the fly photoreceptors.

Specifically, the first-order GFRF, *H*_1_(*jω*) (Fig 2b) can be used to evaluate the magnitude and phase of *Y*_1_(*jω*), the first-order output spectrum of the system, for any single input frequency *U*(*jω*) = |*U*(*jω*)|*e*^*jωt*^ as *Y*_1_(*jω*) = *H*_1_(*jω*)*U*(*jω*).

The second-order GFRF, *H*_2_(*jω*_1_, *jω*_2_) (Fig 2c) can be used to evaluate the magnitude and phase of the second-order output spectrum of the system *Y*_2_(*jω*) at a frequency *ω*, in response to all pairs of input frequencies $U\left(j\omega_{1}\right)\;=\;\left|U\left(j\omega_{1}\right)\right|e^{j\omega_{1}t},$ $U\left(j\omega_{2}\right)\;=\;\left|U\left(j\omega_{2}\right)\right|e^{j\omega_{2}t}$ satisfying *ω* = *ω*_1_ ± *ω*_2_. In general, the *n*^th^-order frequency response function describes the contributions made by combinations of *n* input frequencies to the *n*^th^-order output spectrum. The total output spectrum of the photoreceptor, subject to an arbitrary stimulus, is given by [39].

$$Y\left(j\omega \right)=\sum_{n=1}^{N}Y_{n}\left(j\omega \right)$$ (1)

where *Y*_*n*_(*jω*) is obtained by integrating the contributions from all possible combinations of *n* input frequencies satisfying *ω*_1_ + *ω*_2_ + ⋯ + *ω*_*n*_ = *ω*

$$Y_{n}\left(j\omega \right)=\frac{1}{\sqrt{n}{\left(2\pi \right)}^{n-1}}\int_{\sum_{i=1}^{n}\omega_{i}=\omega }H_{n}\left(j\omega_{1},j\omega_{2},\ldots ,j\omega_{n}\right)\prod_{i=1}^{n}U\left(j\omega_{i}\right)d\sigma_{\omega }$$ (2)

By applying the inverse Fourier transform, we obtain the equivalent time-domain decomposition of the total system response

$$y\left(t\right)=\sum_{n=1}^{N}y_{n}\left(t\right)$$ (3)

into first-order (linear), second- and higher-order responses. Eqs (1–3) provide the key to elucidate the role of nonlinear transformations at the photoreceptor level.

In our case, given a naturalistic stimulus sequence the total response of the photoreceptor ***y*(*t*) = *y***_**0**_ + ***y***_**1**_**(*t*) + h.o.t.** can be approximated just by the first and second-order responses; the relative mean squared error introduced by ignoring the higher order terms is ~1.3% for the bright intensity level *L*_0_ and less than 4E-3 for levels *L*_-1_, *L*_-2_, *L*_-3_. S7g Fig shows *y*(*t*) superimposed on *y*_1_(*t*) +*y*_2_(*t*) whilst S7h Fig shows the magnitude of *Y*_1_(*jω*)+*Y*_2_(*jω*) superimposed on the total output spectrum *Y*(*jω*).

### SNR Computations

The photoreceptor response decomposition derived earlier allows us to evaluate separately the improvement in the Signal-to-Noise Ratio (SNR) of the linear and nonlinear components of the response, relative to the SNR of the noisy stimulus incorporating edges. One would expect that the photoreceptor processing selectively enhances the phase-related measure of feature significance, which are encoded nonlinearly.

Given the input signal

$$u^{s+n}\left(t\right)=u^{s}\left(t\right)+u^{n}\left(t\right)$$

where *u*^*s*^(*t*) is the feature-rich pulse sequence and *u*^*n*^(*t*) is a white noise sequence, the signal-to-noise ratio of *u*(*t*) is defined as

$$SNR\left(u^{s+n}\right)=\frac{P\left(u^{s}\right)}{P\left(u^{n}\right)}$$

where

$$P\left(u^{s,n}\right)=\int_{0}^{T}|u^{s,n}\left(t\right){|}^{2}dt$$

The noise-free photoreceptor output *y*^*s*^(*t*) is the response to the noise-free pulse sequence *u*^*s*^(*t*). The output distortion introduced by the noise is defined

$$y^{n}\left(t\right)=y^{s+n}\left(t\right)-y^{s}\left(t\right)$$

where *y*^*s*+*n*^(*t*) is the response to *u*^*s*+*n*^(*t*).

To characterize the signal-to-noise properties of the first and higher-order responses, we decompose *y*^*s*^(*t*) and *y*^*n*^(*t*) into first and second-order responses and compute

$$SNR\left(y_{1,2}^{s+n}\right)=\frac{P\left(y_{1,2}^{s}\right)}{P\left(y_{1,2}^{n}\right)}$$

The photoreceptor’s noise reduction performance was characterized in terms of the SNR improvement factor

$$Q_{1,2}=M\left\{\frac{SNR\left(y_{1,2}^{s+n}\right)}{SNR\left(u^{s+n}\right)}\right\}$$

which was computed as an average over 50 repetitions. The ‘noise-free’ input *u*^*s*^(*t*) consists of a sequence of positive and negative pulses of amplitude +/-4.75, each lasting 50ms, with a delay of 200ms, superimposed on a constant level of background illumination *L*_0_. To account for the optics of the photoreceptors lens, the pulses were smoothed by convolving the signal with a Gaussian function (5ms standard deviation), accounting for the sensory neurons receptive field properties.

## Results

### Photoreceptor Model Predicts Responses to Arbitrary Stimuli over the Environmental Range of Light Intensities

To demonstrate that the estimated model provides an accurate representation of R1-R6 photoreceptors for the entire environmental range of light intensities, the model was validated extensively using intracellular recordings from the photoreceptors of different flies (S1 File). The model predictions match remarkably well the experimental responses to naturalistic (S4 Fig) and white noise stimuli (S6b Fig) as evidenced by the relative mean squared prediction errors summarized in S1 and S2 Tables respectively. To further illustrate visually the prediction accuracy, we generated an input time series by scanning line by line a naturalistic image (Fig 1d). This temporal stimulus was used to stimulate photoreceptors and measure *in vivo* their responses that were converted back to an image. Fig 1d, shows the original image side-by-side with the images reconstructed from the experimental and model predicted photoreceptor response time series.

### The second-order frequency response function explains the difference in encoding naturalistic and white noise stimuli

To investigate the link between the phase structure of the stimulus and the response, we derived a synthetic stimulus using the magnitude of a Gaussian white noise stimulus and the phase spectrum of a naturalistic stimulus sequence with 1/f magnitude characteristic. The analytical GFRFs derived for our model were used to compute and compare the linear and second-order responses *y*_1_(*t*) and *y*_2_(*t*) to the original Gaussian white noise (GWN) stimulus (Fig 2e top) and to the modified GWN stimulus with naturalistic-phase (Fig 2e bottom).

In line with previous experimental studies [30], we found that **|*Y***_**1**_**(*jω*)|**, the magnitude spectrum of the linear component of the photoreceptor response (Fig 2f–inset panels), dominates the magnitude spectrum of the nonlinear component **|*Y***_**2**_**(*jω*)|** (Fig 2f—main panels) for GWN as well as naturalistic stimuli. However, whilst **|*Y***_**1**_**(*jω*)|** essentially remains unchanged, there is a marked increase of **|*Y***_**2**_**(*jω*)|** for naturalistic stimulus compared to the GWN case (Fig 2f—main panels). This is also reflected in the time domain. The amplitude of the second order component *y*_2_(*t*) of the response to the modified GWN stimulus (Fig 2g bottom) is significantly larger compared to the second order component corresponding to the original GWN stimulus (Fig 2g top). Specifically, whilst the variance of the *y*_2_(*t*) component of the response to the random-phase stimulus represents ~2% of the total response, for the naturalistic-phase stimulus with the same magnitude spectrum, the variance of the *y*_2_(*t*) component increases to ~50% of the variance of the total response.

This increase is entirely due to the non-random structure of the phase spectrum. For the white noise stimulus the Fourier components $U\left(j\omega_{k}\right)\;=\;\left|U\left(j\omega_{k}\right)\right|e^{j\theta_{\omega_{k}}}$ have phase angles $\theta_{\omega_{k}}$ that are uniformly distributed in the range **[0, 2π)** (Fig 2d top). Because the phase of ***H***_**2**_**(*jω***_**1**_, ***jω***_**2**_**)** is remarkably flat for frequencies satisfying ***ω***_***i***_** + *ω***_***j***_ = ***ω*** (see for example the phase slice along *f*_1_+*f*_2_ = *f* = 1 Hz and *f*_1_+*f*_2_ = *f* = 31 Hz shown in Fig 2c), the phase-angles of the ‘weighted input frequencies’ ***H***_**2**_**(*jω***_**1**_, ***jω***_**2**_**)*U*(*jω***_**1**_**)*U*(*jω***_**2**_**)** satisfying ***ω***_***i***_** + *ω***_***j***_ = ***ω*** remain uniformly distributed. Consequently, the magnitude of the second-order frequency spectrum of the response ***Y***_**2**_**(*jω*)**, defined in Eq (2) for *n* = 2, will be small as shown in Fig 2f (top). In essence, the uniform distribution of phases of ***H***_**2**_**(*jω***_**1**_, ***jω***_**2**_**)*U*(*jω***_**1**_**)*U*(*jω***_**2**_**)** means that for every complex vector ***H***_**2**_**(*jω***_**1**_, ***jω***_**2**_**)*U*(*jω***_**1**_**)*U*(*jω***_**2**_**)** with phase *φ* there is a vector ***H***_**2**_**(*jω*′**_**1**_, ***jω*′**_**2**_**)*U*(*jω*′**_**1**_**)*U*(*jω*′**_**2**_**)** with similar amplitude but opposite phase *φ’ = φ*±180; these vector pairs tend to cancel out when the integral Eq (2) is computed. As a result, the corresponding second order temporal response *y*_2_(*t*) is small as seen in Fig 2g (top). The second-order response is not zero because the magnitude of ***H***_**2**_**(*jω***_**1**_, ***jω***_**2**_**)** is not constant along the constant frequency lines ***ω***_***i***_ + ***ω***_***j***_ = ***ω***, as shown in Fig 2c (magnitude slices along *f*_1_+*f*_2_ = 1Hz, for example).

For the modified GWN stimulus with ‘naturalistic’ phase spectrum and white noise magnitude spectrum, the phases of the frequency components ***U*(*jω***_***i***_**)** and the phases of ***H***_**2**_**(*jω***_**i**_, ***jω***_***j***_**)*U*(*jω***_***i***_**)*U*(*jω***_***j***_**)** are not distributed uniformly and as a result, the magnitude of ***Y***_**2**_**(*jω*)** does not cancel out (Fig 2d bottom panels).

The results show that the photoreceptor is sensitive to the phase structure of the temporal stimuli, specifically to correlations between the phases of different frequency components of the temporal stimulus. The analysis shows that the photoreceptor responds linearly to white noise and nonlinearly to naturalistic, feature rich stimuli because of the particular shape of the phase function associated with the second-order frequency response function.

### Fly photoreceptors are tuned to encode robustly temporal local phase congruency

Spatially-localised features, such as edges, are ubiquitous in natural scenes. Edges correlate with contours and textures in a natural scene, which form the basis for higher-level visual processing tasks such as motion detection and object recognition. Not all edge features are characterized by sharp changes in luminance at the boundary of an object. Often edges are blurred or soft-edged like those of shadows [40]. However, all edge-like features exhibit high local phase congruency i.e. the phases of the constituent Fourier components are aligned.

The previous frequency response analysis predicts that points of high local phase congruency, where the difference in phase between different frequency components is zero will elicit very strong second-order responses in fly photoreceptors since in this case the magnitude of ***Y***_**2**_**(*jω*)** is just the integral of the magnitude of ***H***_**2**_**(*jω***_**1**_, ***jω***_**2**_**)*U*(*jω***_**1**_**)*U*(*jω***_**2**_**)** for ***ω***_***i***_** + *ω***_***j***_ = ***ω*** i.e. no cancellation occurs.

To test this hypothesis, we designed a synthetic stimulus consisting of a sequence of narrow square pulses (S1 File) superimposed on a Gaussian white noise sequence with mean *L*_0_ (bright stimulus). The variance of the stimulus was designed such that the output of the automatic gain control module is essentially constant i.e. gain adaptation plays no role in these experiments. The photoreceptor responses to this stimulus, predicted by our model, were found to match closely (S3 Table) the *in vivo* intracellular recordings made from the fly photoreceptors using the same stimulus sequence (S6 Fig).

As before, the model responses were decomposed into linear and nonlinear (second-order) components according to Eqs (1–3) using the GFRFs derived for the NARMAX model with the constant gain corresponding to the ‘bright’ mean intensity level *L*_0_.

**Fig 3:**
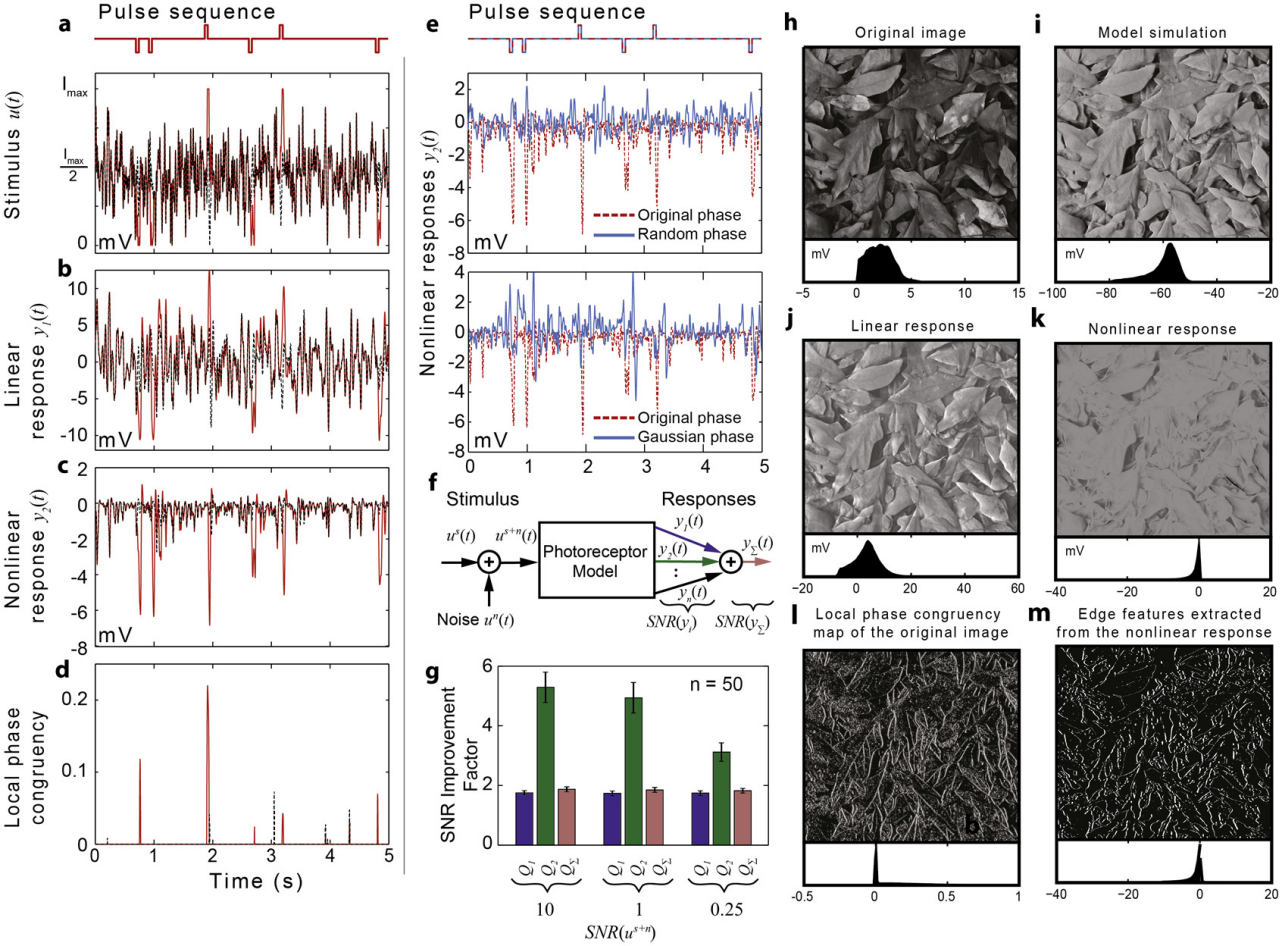
Fly photoreceptors are tuned to detect and enhance phase congruent features. **a**, Synthetic stimulus (red) consisting of a white noise sequence (dashed black line) superimposed by a sequence of square pulses (50 ms duration) (top). **b**,**c**, Linear and nonlinear components of the photoreceptor model responses to the synthetic stimulus (red) and to the pure white noise sequence (dashed black line). **d**, Local phase congruency measure computed for the synthetic (red) and pure white noise stimuli (dashed black line). **e**, Changes to the phase of the second-order frequency response function lead to noisier responses (purple) and makes it almost impossible to locate pulses. **f**, Model used to evaluate SNR improvement factors. **g**, SNR improvement factors calculated for the linear, second-order and total responses, given different levels of input noise. **h**, Input image used to generate light stimulus sequence. **i**, Image reconstructed using the total photoreceptor response. **j**, Image reconstructed using the linear component of the response. **k**, Image reconstructed using the nonlinear component of the response. **l**, Local phase congruency map of the original image. **m**, Edge features extracted by thresholding the nonlinear response.

As seen in (Fig 3a and 3b), the pulses are hard to distinguish from the background noise in the synthetic stimulus and the linear component of the model response. In contrast, the nonlinear component of the response encodes their location quite precisely by large negative peaks (Fig 3c). Even a simple threshold decoder can be used to extract the encoded ‘message’–position of the pulses—from the nonlinear component of the response. A similar decoder applied to the linear response would generate a significant number of false positives.

Remarkably, the nonlinear response appears very similar to the output (Fig 3d) of an established algorithm for computing local phase congruency [15], which is widely used to detect edges in computer vision. Given that the phase congruency measure is invariant to changes in intensity and contrast, it provides arguably the most sparse and efficient representation for edge-and line-like features [14].

A major practical implementation issue is that, being a normalized quantity, phase congruency is highly sensitive to noise. Although the algorithm used to compute phase congruency implements noise reduction techniques [15], it does not detect all the real pulses (Fig 3a) whilst spurious detections still occur (see missing or extra red ‘peaks’ in Fig 3d compared with pulse locations indicated in Fig 3a).

From this point of view, nonlinear transductions at photoreceptor level encode robustly local phase congruence because the nonlinearity is tuned to reject white noise signals. To demonstrate this, we compute the SNR improvement factors (S1 File) for the linear *y*_1_(*t*) and nonlinear *y*_2_(*t*) components of the response to stimuli consisting of a pulse sequence with added white noise having different variance levels. As seen in Fig 3g, for *y*_2_(*t*) the SNR improvement factor *Q*_2_ is significantly higher (almost five fold improvement) than *Q*_1_ computed for *y*_1_(*t*) (two fold improvement).

The nonlinear response encodes robustly the phase correlations buried in noise because the phase of ***H***_**2**_**(*jω***_**1**_, ***jω***_**2**_**)** is almost constant along the integration paths ***ω***_**1**_** + *ω***_**2**_ = ***ω***, which ensures that the phase shift introduced by the second-order frequency response function is independent on the input frequencies.

Artificially changing the phase of the second-order frequency response function, makes the second-order response noisier, reduces the amplitude of the response around the steps and introduces spurious peaks in places where the local phase congruency is low, as seen in Fig 3e. This provides strong evidence that the nonlinear transductions in fly photoreceptors are optimized to enhance behaviourally important higher-order statistical correlations in the natural scenes whilst being largely insensitive to random-phase stimuli.

The reverse-engineered algorithm implemented by photoreceptors is remarkable for its simplicity and, to the best of our knowledge, provides an entirely new approach for computing local phase congruency. While state-of-the art conventional algorithms based on wavelet filter banks are complex, computationally expensive and sensitive to noise [15], the photoreceptor algorithm implements a single nonlinear filtering operation to encode local phase congruency.

To illustrate the practical applicability of the photoreceptor-inspired edge detection algorithm, we computed edge maps for the image shown in Fig 3h by applying a threshold decoder to the standard local phase congruency map of the image (Fig 3h) and to the nonlinear component of the photoreceptor responses to time-series of pixel intensity values along each line of the image. Visually at least, the edge map generated using photoreceptor algorithm (Fig 3m) is ‘cleaner’ than the edge map generated using the standard local phase congruency algorithm (Fig 3l).

### Fly photoreceptors encode non-local phase correlations between the spectral components of the input

**Fig 4:**
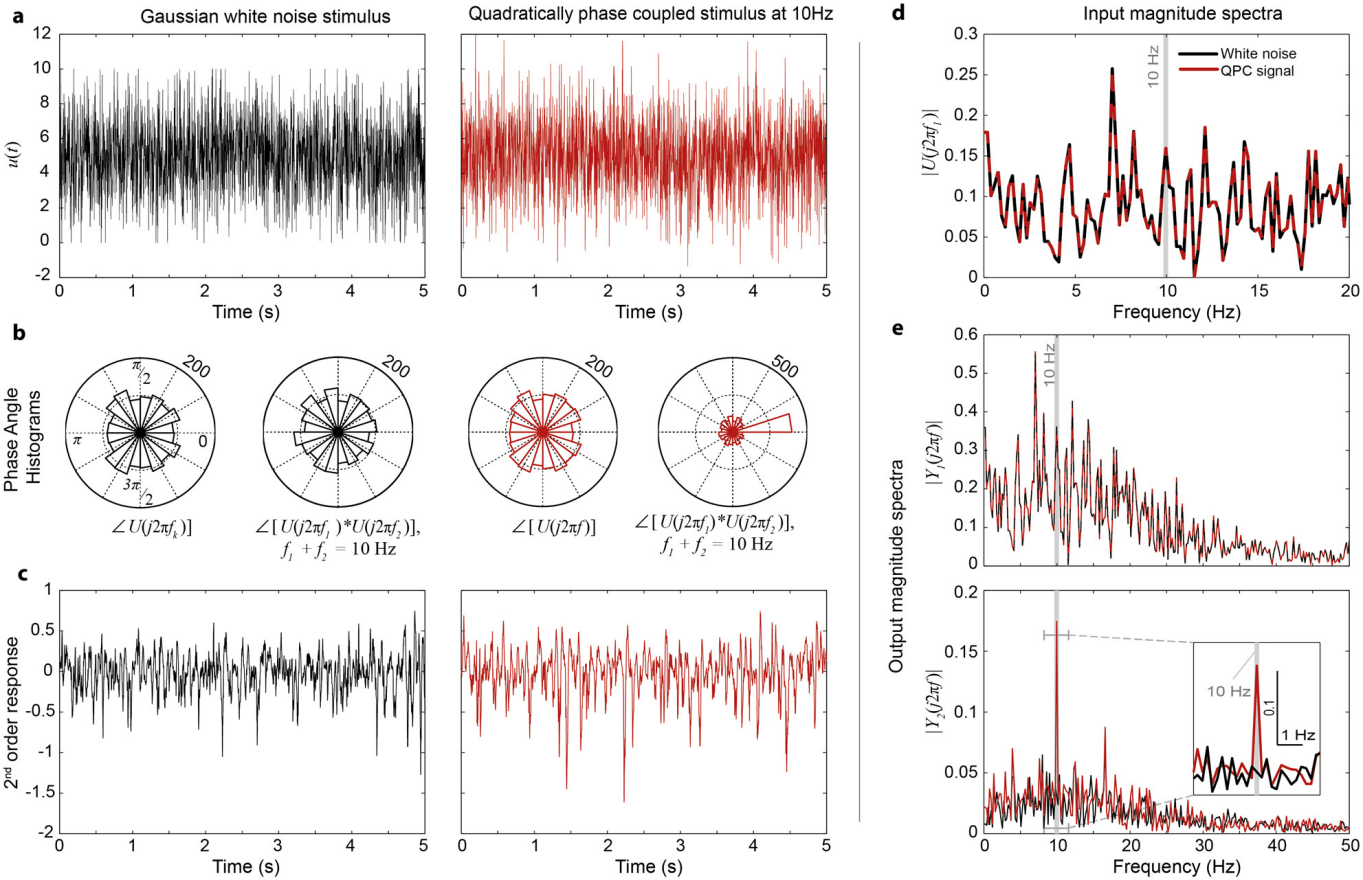
Fly photoreceptors detect quadratic phase coupling. **a**, White noise stimulus (black) and phase-modified white noise stimulus exhibiting quadratic phase coupling (QPC) at 10Hz (red). **b**, Phase angle histograms of the frequency components of the two stimuli. **c**, The second-order component of the model response to the white noise (black) and the QPC (red) stimuli. **d**, Magnitude spectra of the input signals are identical. **e**, While the magnitude spectra of the linear components of the responses are identical, the magnitude spectrum of the second-order component of the response to the QPC stimulus (red) shows significant magnitude increase at 10Hz, compared to the spectrum of the white noise response (black).

Natural images exhibit not only local but also global phase correlations. It has been argued that both local and global higher order statistics of natural images play an important role in texture or symmetry discrimination [41,42]. To test the sensitivity of photoreceptors to non-local phase correlations we designed a synthetic stimulus (Fig 4a), which exhibited quadratic phase coupling (QPC) at 10Hz (i.e. ***phase*(*f***_**1**_**) + *phase*(*f***_**2**_**) = *phase*(*f***_**3**_**)** where ***f***_**1**_ **+ *f***_**2**_ = ***f***_**3**_ = **10** Hz).

Specifically, the QPC stimulus was constructed by computing the Fourier spectrum of a Gaussian white noise signal, modifying the phases of the spectral components to satisfy the above conditions whilst keeping the magnitude function unchanged and finally applying the inverse Fourier transform (S1 File). As seen in Fig 4d, the resulting phase-modified signal has the same Fourier magnitude spectrum as the original white noise signal. However, whilst the phases of the QPC input frequencies ***U*(*jw*)** are still uniformly distributed between 0 and 2π, they are clearly correlated as seen in Fig 4b.

The responses of the photoreceptor model to the white noise and QPC stimuli were decomposed into linear- and second-order responses (Fig 4c). Subsequently, the Fourier spectrum was computed separately for each component of the photoreceptor response.

Because the linear response is not sensitive to the phase structure of the stimulus, the Fourier magnitude spectra of the linear responses to the two stimuli sequences are identical (see Fig 4e and 4b). In contrast, as expected, the second-order response to the QPC stimulus shows a significant increase in the magnitude of the 10 Hz output frequency compared with the second-order response to the white noise stimulus.

### Experimental Validation of the Photoreceptor Model Predictions

The higher order visual processing neurons have to extract the nonlinear codes generated at photoreceptor level from the overall responses. A simple approach to extract the second-order response to a given stimulus is illustrated in S9 Fig. Essentially, the sum of even-order responses to a given stimulus is the average between the photoreceptor response to the stimulus and the response to the out-of-phase (inverted) version of the stimulus. Since in our case the higher-order responses greater than two are negligible, this method generates the second-order component of the photoreceptor response. Using this approach it was possible to demonstrate experimentally that the nonlinear computations performed by the photoreceptor are indeed those predicted by the model-based analysis.

To extract the second-order responses to a given temporal stimulus directly from the experimental recordings we constructed stimulus sequences by alternating the original stimulus sequence with its inverted version (S1 File). The experimental responses to the two versions of the stimuli were averaged and compared with the model predictions. Experiments were carried out using white noise stimuli, stimuli consisting of a pulse sequence superimposed on white noise as well as stimuli exhibiting quadratic phase coupling at 10Hz.

**Fig 5:**
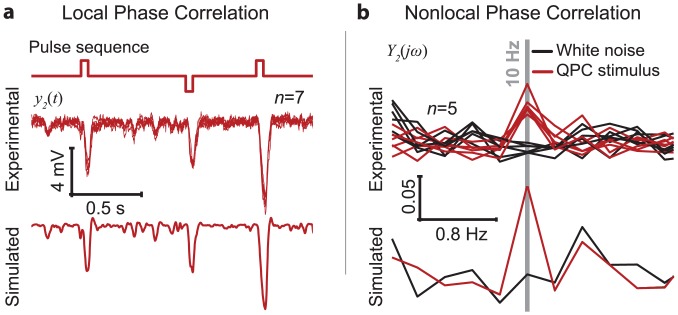
Experimental validation. **a**, The second-order components of the photoreceptor responses extracted directly from *in vivo* recordings (seven repetitions shown) are as predicted by our model. The stimulus used was the pulse sequence superimposed by Gaussian white noise. **b**, The magnitude spectrum of the second-order component of the response to the quadratically phase coupled (QPC) stimulus, extracted directly from in vivo recordings, shows an increase in magnitude at 10Hz as predicted by our model.

Fig 5a shows that the second-order components of the photoreceptor responses (seven repetitions) to the stimulus consisting of the pulse sequence superimposed by Gaussian white noise, extracted directly from the experimental recordings and the model predicted *y*_2_ component.

As predicted, the nonlinear response is significant around points of maximum local phase congruency; the amplitudes of the negative peaks in the nonlinear response at the location of the pulses represent more than 25% of the corresponding peak amplitudes of the total response. The close match between the model predicted and the experimentally derived *y*_2_ component around the negative excursions triggered by the embedded pulses, is further illustrated in S10 Fig. The linear- and the second-order components of the response account for ~91% and ~9% of the overall variance of the total response, respectively. The prediction error variance corresponding to the *y*_2_ component extracted directly from experimental data (S10 Fig) represents ~14% of the total *y*_2_ variance.

On the other hand, the magnitude spectrum of the *y*_2_ component of the photoreceptor response to the QPC stimulus (Fig 5b) shows a ~5 fold increase in magnitude at 10Hz compared to the magnitude spectrum of the *y*_2_ component of the response to the original GWN stimulus, as predicted by model.

Given that the photoreceptor model was derived using experimental recordings from a different fly, these results demonstrate further the validity of our model and, more importantly, that the nonlinear encoding of phase correlations is a generic information processing strategy of fly photoreceptors.

### Investigating the role of the retinal network

Photoreceptor responses in wild flies are modulated by feedback from two classes of interneurons, i.e. large monopolar cells (LMC) and amacrine cells (AC), and from axonal gap-junctions, which pool the responses from six photoreceptors [43–45]. It is therefore natural to ask to what extent the processing capabilities demonstrated earlier are due to temporal processing by the photoreceptor alone. To elucidate this question, we measured photoreceptor responses to the original multi-level naturalistic stimulus in blind *hdc*^JK910^ mutants [46,47] that lack histamine in their photoreceptors. Fly photoreceptors use neurotransmitter histamine to communicate visual information to interneurons [48]. In the histamine deficient mutants, the lamina interneurons fail to receive and transmit visual information and their feedback synapses can no longer modulate photoreceptor output [47]. Essentially, these mutant flies are blind. By comparing intracellular recordings from photoreceptors of wild type flies to those of *hdc*^JK910^ mutants, one can test how the lamina network affects adaptation and information processing in photoreceptors.

**Fig 6:**
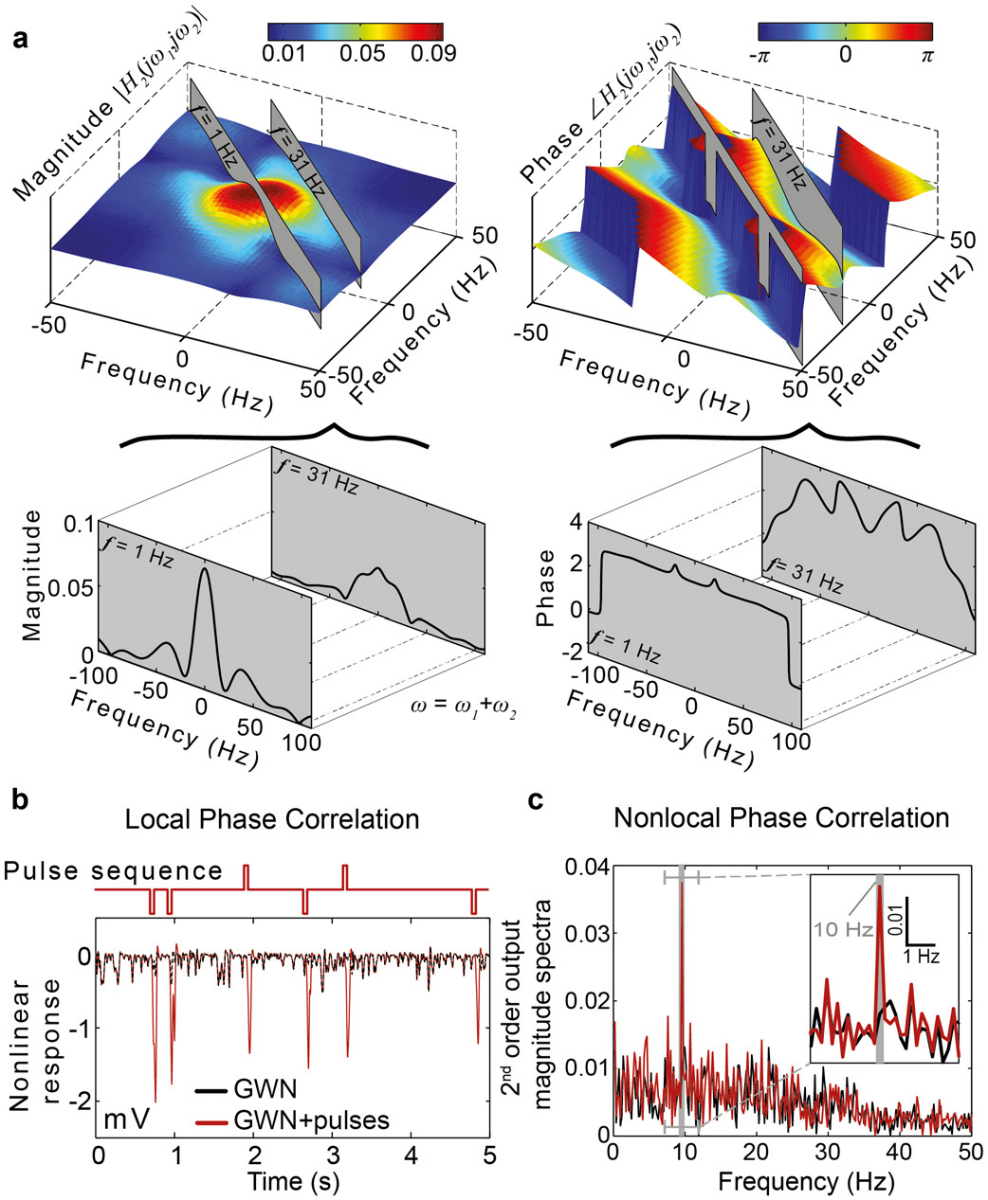
Isolated photoreceptors in the *hdc*^JK910^ mutant fly demonstrate similar phase processing capabilities to those of wild-type flies. **a**, The second-order frequency response functions computed for photoreceptors of *hdc*^JK910^ mutants are similar to those computed for wild-type photoreceptors. Nonlinear component of the *hdc*^JK910^ photoreceptor model response clearly indicate location of the 50 ms pulses. **c**, The magnitude spectrum of the second-order response to the quadratically phase coupled stimulus shows increase in magnitude at 10Hz.

As seen in S11 Fig, mutant photoreceptor responses have dramatically reduced contrast sensitivity for bright (*L*_0_) stimuli and their capability to quickly adapt to the mean illumination is significantly impaired. However, the light adapted responses of histamine photoreceptors to naturalistic stimuli having a mean luminance *L-*_1_ are very similar to wild-type responses. As we are interested to investigate the nonlinear properties of isolated photoreceptors exhibiting a normal response range, we inferred and validated (S12 Fig) a mutant photoreceptor model using measured responses to naturalistic stimuli sequences with mean luminance *L-*_1_ (S1 File). The second-order GFRFs (Fig 6a), derived for this model, are very similar to those of wild-type photoreceptors. In particular, the phase constancy along integration lines is preserved in mutant flies. As a consequence, the mutant photoreceptor responses to the two classes of synthetic stimuli are very similar to the wild type responses (Fig 6b), providing strong evidence that the nonlinear transformations, underlying the detection of high-order phase correlation in the temporal light patterns, are performed by the photoreceptor alone, independently of neighbouring neurons.

## Discussion

We have demonstrated that R1-R6 photoreceptors perform nonlinear transformations that encode biologically relevant, higher-order statistical features that are represented in the Fourier phase spectrum of temporal stimuli. In particular, we have shown that photoreceptors encode points of maximum local phase congruency, which occur at the location of an edge or line, as well as long-range phase correlations, which characterize symmetry and texture properties of natural images [41].

An important conclusion of our analysis is that the nonlinear transductions in fly photoreceptors are tuned to maximize the response to combinations of spectral components that are congruent in phase or are phase-coupled and to minimize the response to temporal stimuli with a random phase spectrum. This ensures that the nonlinear coding is robust to noise and explains why photoreceptors respond linearly to non-informative white noise stimuli and nonlinearly to naturalistic time-series exhibiting local and global phase correlations between their spectral components. It also explains why the models derived using responses to white noise stimuli fail to capture the key nonlinear transformations performed by photoreceptors.

This strategy for processing temporal stimuli does not require dynamic adaptation to stimuli statistics beyond the mean and variance. Specifically, we show that what appears to be an adaptation of the photoreceptor to the statistical structure of the stimuli is in fact explained by the shape of the two-dimensional phase function corresponding to the second-order GFRF of the photoreceptor, which is almost constant along the lines ***ω***_***i***_ **+ *ω***_***j***_ = ***constant***.

From the point of view of information theory, different encoding of naturalistic and white noise signals can be viewed as a solution to the problem of matching the stimulus (source) with the communication channel in a probabilistic sense in order to achieve an optimal trade-off between two competing goals: minimizing distortion in decoding behaviourally relevant stimuli features and minimizing the information rate, that is, the energetic costs associated with phototransduction [49].

The fact that the second-order phase function computed for a separate photoreceptor model, derived for *Caliphora*, exhibits similar characteristics (see S8 Fig) to those of *Drosophila*, suggests that the maximization of sensitivity to phase aligned or coupled frequency components is a fundamental ‘design principle’ of fly photoreceptors, which may well apply to other sensory neurons. Previous studies of the primary visual cortex [42,50] have postulated the presence of nonlinear mechanisms that are sensitive to phase correlations. Here we demonstrate for the first time that in *Drosophila* these mechanisms operate at the photoreceptor level. The main benefit of implementing nonlinear encoding at photoreceptor level is that it may facilitate the efficient decoding of key stimuli features such as edges, at higher visual processing levels. Essentially, as we have illustrated earlier, edge maps could easily be extracted from the nonlinear component of the response using a simple threshold decoder. Given that neural circuits are highly optimized, one would expect that in the absence of the nonlinear photoreceptor code the higher visual processing stages would have to implement additional computations, leading to more complex downstream neural circuit architectures. At the same time, encoding temporal edges at photoreceptor level, before the information from six photoreceptors has been pooled by the interneurons, should help improve further the signal-to-noise ratio of the encoded features.

The similarities that exist between responses of primate cones and blowfly photoreceptors [51] suggest that nonlinear transformations performed by cone photoreceptors ultimately achieve the same processing goals, albeit using different molecular mechanisms and signal processing steps.

Previous experimental and theoretical studies of early visual processing in humans indicate the existence of detectors that are highly sensitive to features characterized by high phase congruency [52,53]. We speculate that human photoreceptors implement similar nonlinear processing of the visual stimuli to detect phase congruency, which could help explain why neurons in the primary visual cortex can reliably signal phase congruence and how the phase congruency information is extracted from the visual stimuli. Since moving spatial edges generated by saccadic eye movements or by moving objects generate temporal edges at photoreceptor level, selectively encoding these temporal features and enhancing their salience, should facilitate downstream processing of the spatio-temporal visual stimuli for edge and motion detection [54].

The simple technique we used to separate the second-order response directly from experimental recordings could easily be used to test this hypothesis for mammalian retinal cones.

One could envisage a simple model where eye saccades map localized spatial edges onto temporal edges that are encoded and enhanced by photoreceptors, enabling the downstream neural circuits to use timing in addition to spatial information to detect edges and group them into contours.

As it is not clear how the higher-order spiking neurons could implement efficiently this processing step, it could be that all downstream visual processing relies on the phase congruency information generated by photoreceptors to the extent that the absence of this information may incapacitate downstream feature detectors. If this were true, applying such nonlinear transformations to the visual stimuli, prior to delivering these to retinal or ganglion cells, may improve significantly the performance of artificial retinas [55,56].

## Supporting Information

S1 Fig Experimental setup for the acquisition of *in-vivo* intracellular photoreceptor responses to light stimuli patterns. (TIF)

S2 Fig Naturalistic stimuli. **a**, 2 s representative naturalistic stimulus sequence, used for the modelling procedure. **b**, Typical 1/f power spectrum of a naturalistic stimulus. **c**, Naturalistic input sequence and corresponding photoreceptor response. The grey arrows indicate the transient responses during adaptation. **d**, Adaptation to different duration of stationary stimuli. On the tested timescales (2 s, black; 4 s, purple; 8 s, green) adaptation dynamics are little dependent on the length of the stationary stimulus.
(TIF)

S3 Fig Neural responses at stationary regimes. **a**, Experimental transient (grey) and stationary (black) responses to NTSI stimuli with instant light changes. Slow transients and trends in stationary regions were removed by polynomial fittings (red traces). **b**, Photoreceptor responses to repeated stimuli sequences for different mean intensity levels (black) and average response (red) over the 16 s intervals highlighted.
(TIF)

S4 Fig Comparison of neural responses of different animals. The average photoreceptor response of the six flies for different mean light intensity levels after normalization to a common response offset and deviation.
(TIF)

S5 Fig Estimation and validation of the gain control model. **a**, Steady state gain (black) vs. mean light intensity (brown). **b**, Block diagram of the photoreceptor model incorporating the gain control law (dashed-line box). **c**, Multilevel light contrast stimulus. **d**, Gain control model predictions (red) superimposed on the estimated gain response (black).
(TIF)

S6 Fig Model validation using data recorded from different wild type fly photoreceptors at light level *L*_0_. **a**, Normalized model predictions to a naturalistic stimulus data sequence compared with the average response (n = 8) of 4 cells. **b**, as in **a** but for a 100 Hz band limited white noise stimulus sequence for 3 cells. **c**, as in **a** but for a stimulus with positive and negative pulses embedded in noise measured in 8 different photoreceptors. Gain response. The box plots are used to show the variations across experimental recordings prior to normalization.
(TIF)

S7 Fig Model response decomposition. **a**, Naturalistic input sequence. **b**, Input spectrum. **c**, Linear component of the response. **d**, First-order output frequency response. **e**, Second-order component of the output. **f**, Second-order output frequency response. **g**, Combined first- and second-order time responses (red) match almost perfectly the overall model response (black). **h**, the combined first- and second-order output frequency response (red) matches the overall output frequency spectrum (black).
(TIF)

S8 Fig Second-order phase functions (slices), shown along integration lines of constant frequency, computed for a *Calliphora* photoreceptor model. (TIF)

S9 Fig **a**, Computation of even-order responses. **b**, Synthetic light stimulus consisting of a sequence of square pulses superimposed on a white noise sequence. **c**, Inverted version of the stimulus given in **b**. **d** and **e**, Photoreceptor model (mean subtracted) responses to the stimuli given in **b** and **c** respectively. **f**, Even-order response computed by averaging the model predictions shown in **d** and **e**, (red) and model predicted nonlinear response (black). **g**, Local phase congruency measure computed for the synthetic stimulus.
(TIF)

S10 Fig Nonlinear component of the response *y*_2_(*t*) predicted by the model (dash-dotted red line) vs. *y*_2_(*t*) component extracted directly from experimental data (solid black line). (TIF)

S11 Fig Experimental recordings in photoreceptors of histamine mutants. **a**, ERG and voltage responses measured in photoreceptors of wild-type (black line), *hdc*^JK910^ mutant (red line) and rescued *hdc*^JK910^ mutant (grey line) flies. ERG voltage responses of histamine deficient *hdc*^JK910^ mutants lack on-off transients (red arrows, middle panel), demonstrating that synaptic communication between photoreceptors and lamina interneurons is interrupted. The voltage responses (right panel) suggest that synaptic communication increases the range of environmental light intensities to which R1‐R6 photoreceptors can adapt. Arrows highlight the key differences in mutant photoreceptor responses compared to wild-type responses: contrast saturation for bright stimuli and impaired dynamic adaptation. Both wild‐type and the histamine rescued photoreceptors show normal processing of naturalistic contrast pattern stimuli. **b**, Whole-cell patch-clamp recordings of current responses to 1s prolonged light and flash light stimuli and to 1s Voltage steps in dissociated *hdc*^JK910^ photoreceptors.
(TIF)

S12 Fig Modelling the photoreceptor responses of the histamine mutant flies. **a**, Boxplots of response amplitudes during stationary light stimulation at light level *L*_-1_ for 5 different flies. **b**, The mean response based of 8 responses to a single naturalistic stimulus sequence. The mean responses of individual flies are normalized to the mean deviation and amplitude of all flies tested. **c**, Experimentally measured responses to a repeated stimulus (data set #5) which were used to infer a photoreceptor model. **d**, Prediction performance of the *hdc*^JK910^ photoreceptor model. **e**, Correlation tests.
(TIF)

S1 File Supplementary Methods. (PDF)

S1 Table Relative mean square prediction error calculated using the model predicted output and normalized photoreceptor responses, measured in six flies (S4 Fig), to the naturalistic stimuli with different mean light intensity levels. (DOCX)

S2 Table Relative mean square prediction error calculated using the model predicted output and normalized photoreceptor responses, measured in three flies (level *L*_0_ responses shown in S6b Fig), to bandlimited (100 Hz) white noise stimuli with different mean intensity levels. (DOCX)

S3 Table Relative mean square prediction error calculated using the model predicted output and normalized photoreceptor responses, measured in eight flies (S6c Fig), to the GWN+pulses stimulus sequence corresponding to a mean light intensity level *L*_0_. (DOCX)

## References

[1] Barlow HBH. Possible principles underlying the transformation of sensory messages In: Rosenblith W, editor. Sensory Communication. MIT Press; 1961 pp. 217–234.

[2] Barlow H. Redundancy reduction revisited. Netw Comput Neural Syst. 2001;12: 241–253. 10.1080/net.12.3.241.253 11563528

[3] Victor JD, Shapley RM. Receptive field mechanisms of cat X and Y retinal ganglion cells. J Gen Physiol. 1979;74: 275–98. Available: http://www.ncbi.nlm.nih.gov/pubmed/490143 490143 10.1085/jgp.74.2.275 PMC2228497

[4] Rieke F, Bodnar DA, Bialek W. Naturalistic stimuli increase the rate and efficiency of information transmission by primary auditory afferents. Proc Biol Sci. 1995;262: 259–65. 10.1098/rspb.1995.0204 8587884

[5] Theunissen FE, Sen K, Doupe AJ. Spectral-temporal receptive fields of nonlinear auditory neurons obtained using natural sounds. J Neurosci. 2000;20: 2315–31. Available: http://www.ncbi.nlm.nih.gov/pubmed/10704507 10704507 10.1523/JNEUROSCI.20-06-02315.2000 PMC6772498

[6] Schneider DM, Woolley SMN. Extra-Classical Tuning Predicts Stimulus-Dependent Receptive Fields in Auditory Neurons. J Neurosci. 2011;31: 11867–11878. 10.1523/JNEUROSCI.5790-10.2011 21849547 PMC3164972

[7] Vickers NJ, Christensen TA, Baker TC, Hildebrand JG. Odour-plume dynamics influence the brain’s olfactory code. Nature. 2001;410: 466–70. 10.1038/35068559 11260713

[8] van Hateren JH, Snippe HP. Information theoretical evaluation of parametric models of gain control in blowfly photoreceptor cells. Vision Res. 2001;41: 1851–65. Available: http://www.ncbi.nlm.nih.gov/pubmed/11369048 11369048 10.1016/s0042-6989(01)00052-9

[9] Juusola M, de Polavieja GG. The rate of information transfer of naturalistic stimulation by graded potentials. J Gen Physiol. 2003;122: 191–206. 10.1085/jgp.200308824 12860926 PMC2229540

[10] Sharpee TO, Sugihara H, Kurgansky A V., Rebrik SP, Stryker MP, Miller KD. Adaptive filtering enhances information transmission in visual cortex. Nature. 2006;439: 936–942. 10.1038/nature04519 16495990 PMC2562720

[11] Carandini M, Demb JB, Mante V, Tolhurst DJ, Dan Y, Olshausen BA, et al Do we know what the early visual system does? J Neurosci. 2005;25: 10577–10597. 10.1523/JNEUROSCI.3726-05.2005 16291931 PMC6725861

[12] Simoncelli EP, Olshausen BA. Natural image statistics and neural representation. 2001; 10.1146/annurev.neuro.24.1.1193 11520932

[13] Morrone MC, Owens RA. Feature detection from local energy. Pattern Recognit Lett. 1987;6: 303–313.

[14] Oppenheim AV, Lim JS. The importance of phase in signals. Proc IEEE. 1981;69: 529–541. 10.1109/PROC.1981.12022

[15] Kovesi P. Image features from phase congruency. Videre J Comput Vis Res. 1999;1: 1–27.

[16] Brinkworth RSA, Mah EL, Gray JP, O’Carroll DC, F. A, A. BS, et al Photoreceptor processing improves salience facilitating small target detection in cluttered scenes. J Vis. The Association for Research in Vision and Ophthalmology; 2008;8: 8–8. 10.1167/8.11.8 18831602

[17] Thomson MG. Visual coding and the phase structure of natural scenes. Network. 1999;10: 123–32. Available: http://www.ncbi.nlm.nih.gov/pubmed/10378188 10378188

[18] Juusola M, Kouvalainen E, Järvilehto M, Weckström M. Contrast gain, signal-to-noise ratio, and linearity in light-adapted blowfly photoreceptors. J Gen Physiol. 1994;104: 593–621. Available: http://www.ncbi.nlm.nih.gov/pubmed/7807062 7807062 10.1085/jgp.104.3.593 PMC2229225

[19] Marr D. Vision a computational investigation into the human representation and processing of visual information. MIT Press; 2010.

[20] Ruderman DL, Bialek W. Statistics of Natural Scenes: Scaling in the Woods. Phys Rev Lett. 1994;73: 814–817. 10.1038/1811181a0 10057546

[21] Olshausen BA, Field DJ. Natural image statistics and efficient coding. Netw Comput Neural Syst. 1996;7: 333–339. 10.1088/0954-898X_7_2_014 16754394

[22] Tkačik G, Prentice JS, Victor JD, Balasubramanian V. Local statistics in natural scenes predict the saliency of synthetic textures. Proc Natl Acad Sci. 2010;107: 18149–18154. 10.1073/pnas.0914916107 20923876 PMC2964243

[23] Field DJ. Relations between the statistics of natural images and the response properties of cortical cells. J Opt Soc Am A. 1987;4: 2379 10.1364/JOSAA.4.002379 3430225

[24] Wainwright MJ, Schwartz O, Simoncelli EP. Natural image statistics and divisive normalization: Modeling nonlinearities and adaptation in cortical neurons. Statistical theories of the brain. 2001 pp. 203–222.

[25] Srinivasan M V., Bernard GD. The effect of motion on visual acuity of the compound eye: A theoretical analysis. Vision Res. 1975;15: 515–525. 10.1016/0042-6989(75)90029-2 1129998

[26] Srinivasan M V., Bernard GD. The pursuit response of the housefly and its interaction with the optomotor response. J Comp Physiol A. 1977;115: 101–117. 10.1007/BF00667788

[27] Juusola M, French AS, Egelhaaf M, Hausen K, Reichardt W, Wehrhahn C, et al Visual acuity for moving objects in first- and second-order neurons of the fly compound eye. J Neurophysiol. 1997;77: 1487–95. 9084613 10.1152/jn.1997.77.3.1487

[28] Burton BG, Laughlin SB. Neural images of pursuit targets in the photoreceptor arrays of male and female houseflies Musca domestica. J Exp Biol. 2003;206: 3963–77. 10.1242/jeb.00600 14555737

[29] Van Hateren JH. Processing of Natural Time Series of Intensities by the Visual System of the Blowfly. Vis Res. 1997;37: 3407–3416. 9425553 10.1016/s0042-6989(97)00105-3

[30] Juusola M, Hardie RC. Light adaptation in Drosophila photoreceptors: I. Response dynamics and signaling efficiency at 25 degrees C. J Gen Physiol. 2001;117: 3–25. 11134228 10.1085/jgp.117.1.3 PMC2232468

[31] Chen S, Billings SA. Representations of non-linear systems: the NARMAX model. Int J Control. 1989;49: 1013–1032. 10.1080/00207178908559683

[32] Billings SA. Nonlinear system identification. Chichester, UK: John Wiley & Sons, Ltd; 2013.

[33] Song Z, Postma M, Billings SA, Coca D, Hardie RC, Juusola M. Stochastic, adaptive sampling of information by microvilli in fly photoreceptors. Curr Biol. 2012;22: 1371–1380. 10.1016/j.cub.2012.05.047 22704990 PMC3420010

[34] French AS, Korenberg MJ, Järvilehto M, Kouvalainen E, Juusola M, Weckström M. The dynamic nonlinear behavior of fly photoreceptors evoked by a wide range of light intensities. Biophys J. 1993;65: 832–9. 10.1016/S0006-3495(93)81116-0 8218908 PMC1225784

[35] Billings SA, Tsang KM. Spectral analysis for non-linear systems, Part II: Interpretation of non-linear frequency response functions. Mech Syst Signal Process. 1989;3: 341–359.

[36] Billings SA, Peyton Jones JC. Mapping non-linear integro-differential equations into the frequency domain. Int J Control. 1990;52: 863–879. 10.1080/00207179008953572

[37] Jones JCP, Billings SA. Recursive algorithm for computing the frequency response of a class of non-linear difference equation models. Int J Control. 1989;50: 1925–1940. 10.1080/00207178908953474

[38] Asyali MH, Juusola M. Use of Meixner functions in estimation of Volterra kernels of nonlinear systems with delay. IEEE Trans Biomed Eng. 2005;52: 229–237. 10.1109/TBME.2004.840187 15709660

[39] Lang ZQ, Billings SA, Yue R, Li J. Output frequency response function of nonlinear Volterra systems. Automatica. 2007;43: 805–816. 10.1016/j.automatica.2006.11.013

[40] Morrone MC, Burr DC. Feature detection in human vision: a phase-dependent energy model. Proc R Soc London Ser B, Biol Sci. 1988;235: 221–45. 10.1098/rspb.1988.0073 2907382

[41] Oka S, Victor JD, Conte MM, Yanagida T. VEPs elicited by local correlations and global symmetry: Characteristics and interactions. Vision Res. 2007;47: 2212–2222. 10.1016/j.visres.2007.03.020 17604074 PMC2041857

[42] Victor JD, Mechler F, Ohiorhenuan I, Schmid AM, Purpura KP, Abbott L, et al Laminar and orientation-dependent characteristics of spatial nonlinearities: implications for the computational architecture of visual cortex. J Neurophysiol. 2009;102: 3414–32. 10.1152/jn.00086.2009 19812295 PMC2804422

[43] Zheng L, Nikolaev A, Wardill TJ, O’Kane CJ, de Polavieja GG, Juusola M. Network adaptation improves temporal representation of naturalistic stimuli in Drosophila eye: I dynamics. PLoS One. 2009;4: e4307 10.1371/journal.pone.0004307 19180196 PMC2628724

[44] Shaw SR. Early visual processing in insects. J Exp Biol. 1984;112: 225–51. Available: http://www.ncbi.nlm.nih.gov/pubmed/6392468 6392468 10.1242/jeb.112.1.225

[45] Wardill TJ, List O, Li X, Dongre S, McCulloch M, Ting C-Y, et al Multiple spectral inputs improve motion discrimination in the Drosophila visual system. Science. 2012;336: 925–31. 10.1126/science.1215317 22605779 PMC6528803

[46] Burg MG, Sarthy P V, Koliantz G, Pak WL. Genetic and molecular identification of a Drosophila histidine decarboxylase gene required in photoreceptor transmitter synthesis. EMBO J. 1993;12: 911–9. Available: http://www.ncbi.nlm.nih.gov/pubmed/8096176 8096176 10.1002/j.1460-2075.1993.tb05732.x PMC413291

[47] Melzig J, Buchner S, Wiebel F, Wolf R, Buchner E, Burg M, et al Genetic depletion of histamine from the nervous system of Drosophila eliminates specific visual and mechanosensory behavior. J Comp Physiol A. 1996;179: 763–773. 10.1007/BF00207355 8956497

[48] Hardie RC. A histamine-activated chloride channel involved in neurotransmission at a photoreceptor synapse. Nature. 1989;339: 704–706. 10.1038/339704a0 2472552

[49] Niven JE, Anderson JC, Laughlin SB. Fly photoreceptors demonstrate energy-information trade-offs in neural coding. PLoS Biol. 2007;5: e116 10.1371/journal.pbio.0050116 17373859 PMC1828148

[50] Felsen G, Touryan J, Han F, Dan Y. Cortical sensitivity to visual features in natural scenes. PLoS Biol. 2005;3: e342 10.1371/journal.pbio.0030342 16171408 PMC1233414

[51] van Hateren JH, Snippe HP. Phototransduction in primate cones and blowfly photoreceptors: different mechanisms, different algorithms, similar response. J Comp Physiol A. 2006;192: 187–197. 10.1007/s00359-005-0060-y 16249881

[52] Mechler F, Reich DS, Victor JD. Detection and discrimination of relative spatial phase by V1 neurons. J Neurosci. 2002;22: 6129–57. 12122074 10.1523/JNEUROSCI.22-14-06129.2002 PMC6757932

[53] Henriksson L, Hyvarinen A, Vanni S. Representation of cross-frequency spatial phase relationships in human visual cortex. J Neurosci. 2009;29: 14342–14351. 10.1523/JNEUROSCI.3136-09.2009 19906981 PMC6665080

[54] Rucci M, Iovin R, Poletti M, Santini F. Miniature eye movements enhance fine spatial detail. Nature. 2007;447: 852–855. 10.1038/nature05866 17568745

[55] Mathieson K, Loudin J, Goetz G, Huie P, Wang L, Kamins TI, et al Photovoltaic retinal prosthesis with high pixel density. Nat Photonics. 2012;6: 391–397. 10.1038/nphoton.2012.104 23049619 PMC3462820

[56] Zrenner E, Bartz-Schmidt KU, Benav H, Besch D, Bruckmann A, Gabel V-P, et al Subretinal electronic chips allow blind patients to read letters and combine them to words. Proc Biol Sci. 2011;278: 1489–97. 10.1098/rspb.2010.1747 21047851 PMC3081743

