# Supplementary figures and images for: Fly Photoreceptors Encode Phase Congruency
Source: PLoS One. 2016 Jun 23;11(6):e0157993. doi: 10.1371/journal.pone.0157993 (PMC4919002; doi:10.1371/journal.pone.0157993)

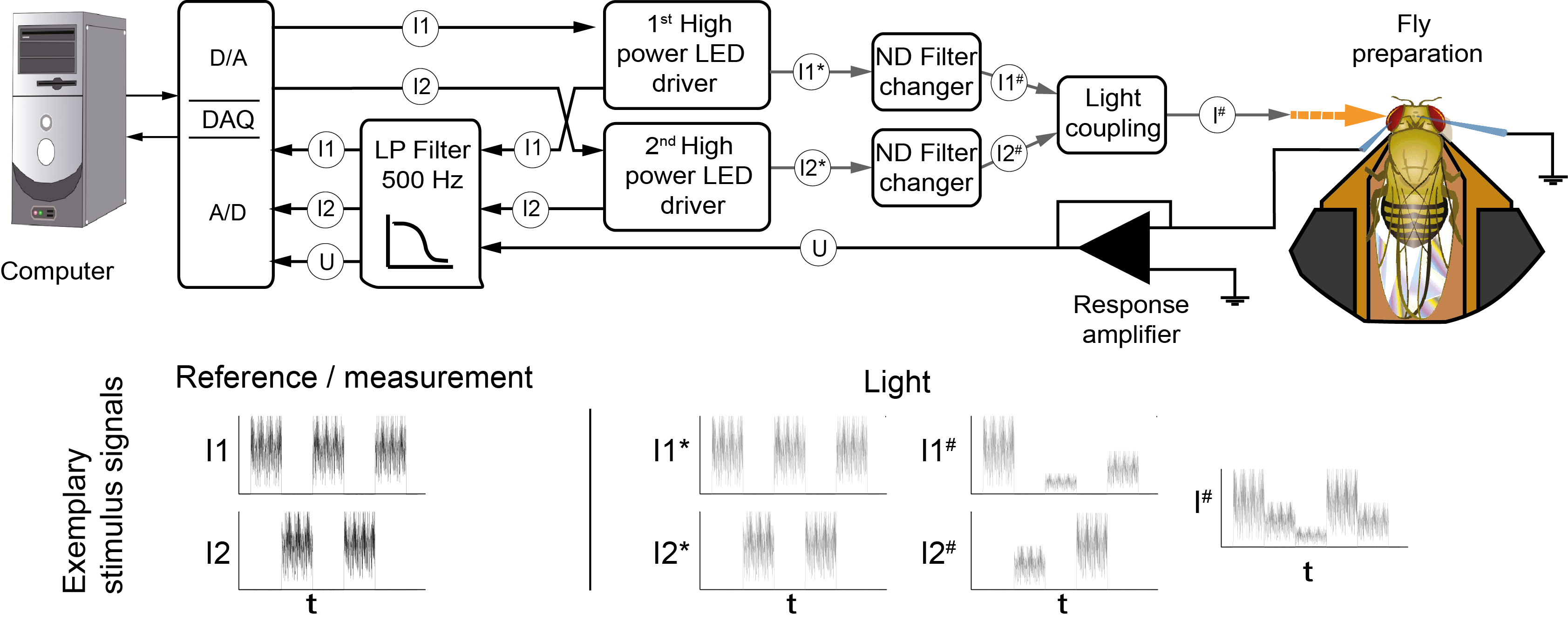

Supplement: S1 Fig — (TIF) [file pone.0157993.s001.tif]

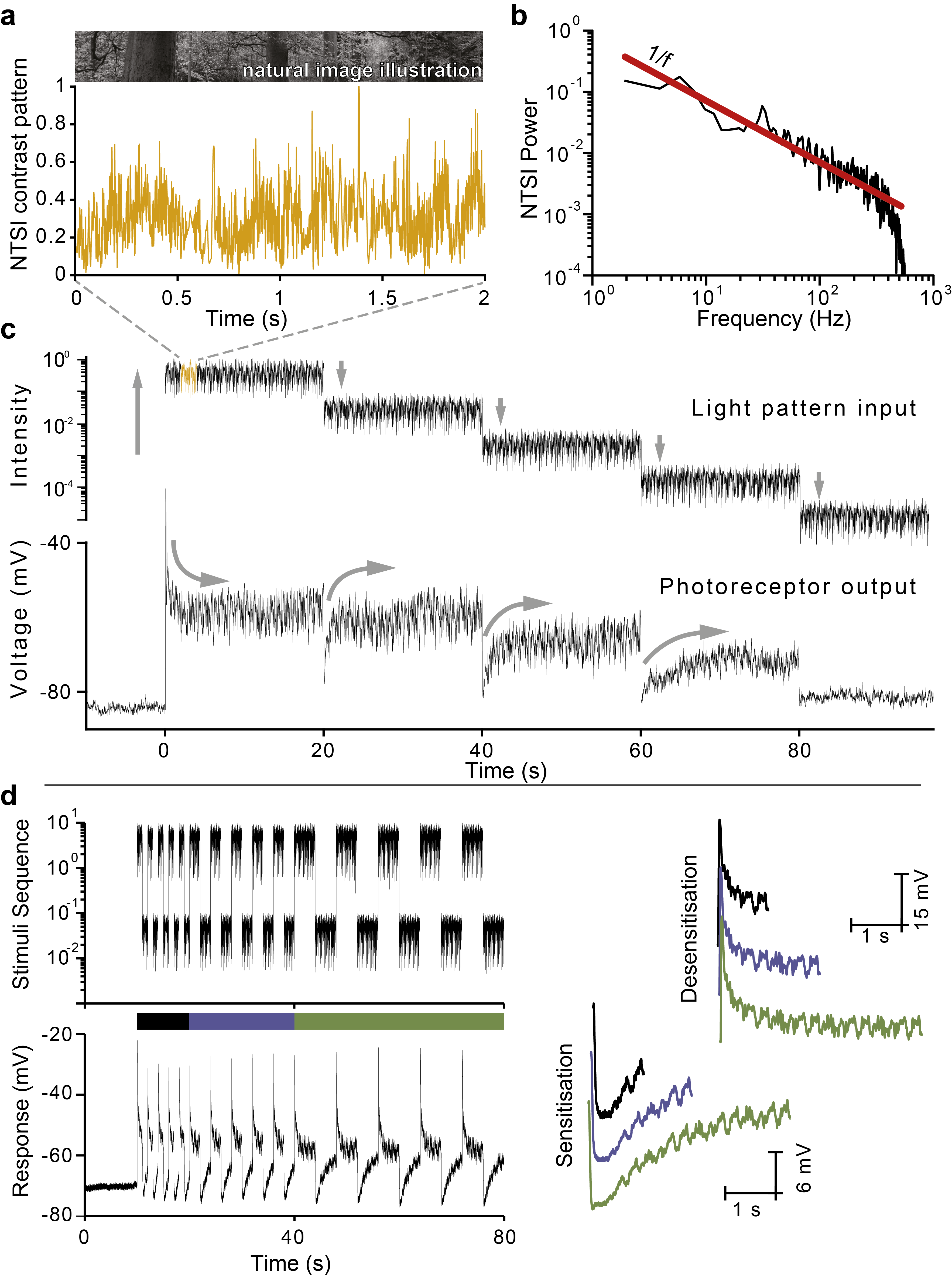

Supplement: S2 Fig — a, 2 s representative naturalistic stimulus sequence, used for the modelling procedure. b, Typical 1/f power spectrum of a naturalistic stimulus. c, Naturalistic input sequence and corresponding photoreceptor response. The grey arrows indicate the transient responses during adaptation. d, Adaptation to different duration of stationary stimuli. On the tested timescales (2 s, black; 4 s, purple; 8 s, green) adaptation dynamics are little dependent on the length of the stationary stimulus. (TIF) [file pone.0157993.s002.tif]

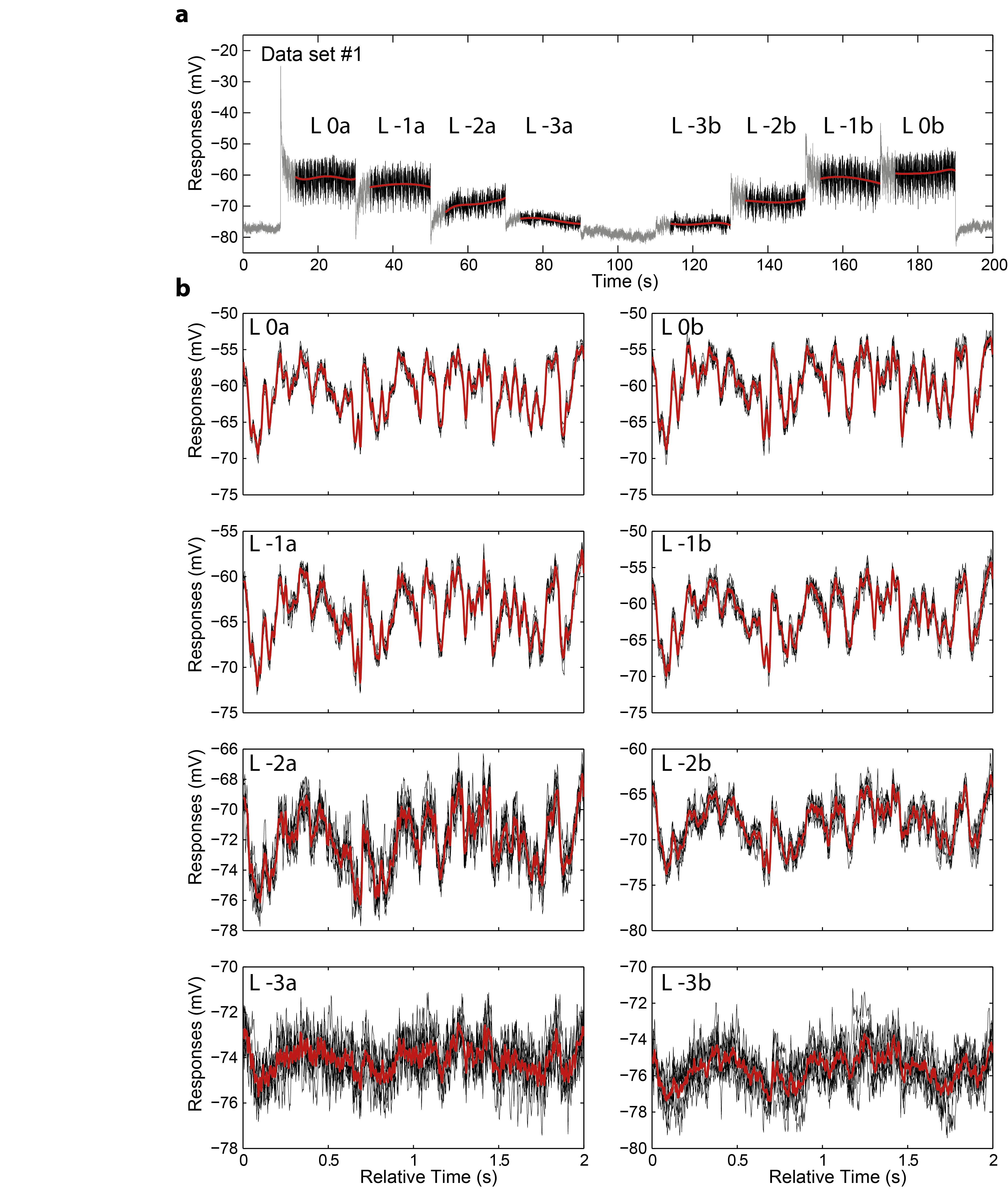

Supplement: S3 Fig — a, Experimental transient (grey) and stationary (black) responses to NTSI stimuli with instant light changes. Slow transients and trends in stationary regions were removed by polynomial fittings (red traces). b, Photoreceptor responses to repeated stimuli sequences for different mean intensity levels (black) and average response (red) over the 16 s intervals highlighted. (TIF) [file pone.0157993.s003.tif]

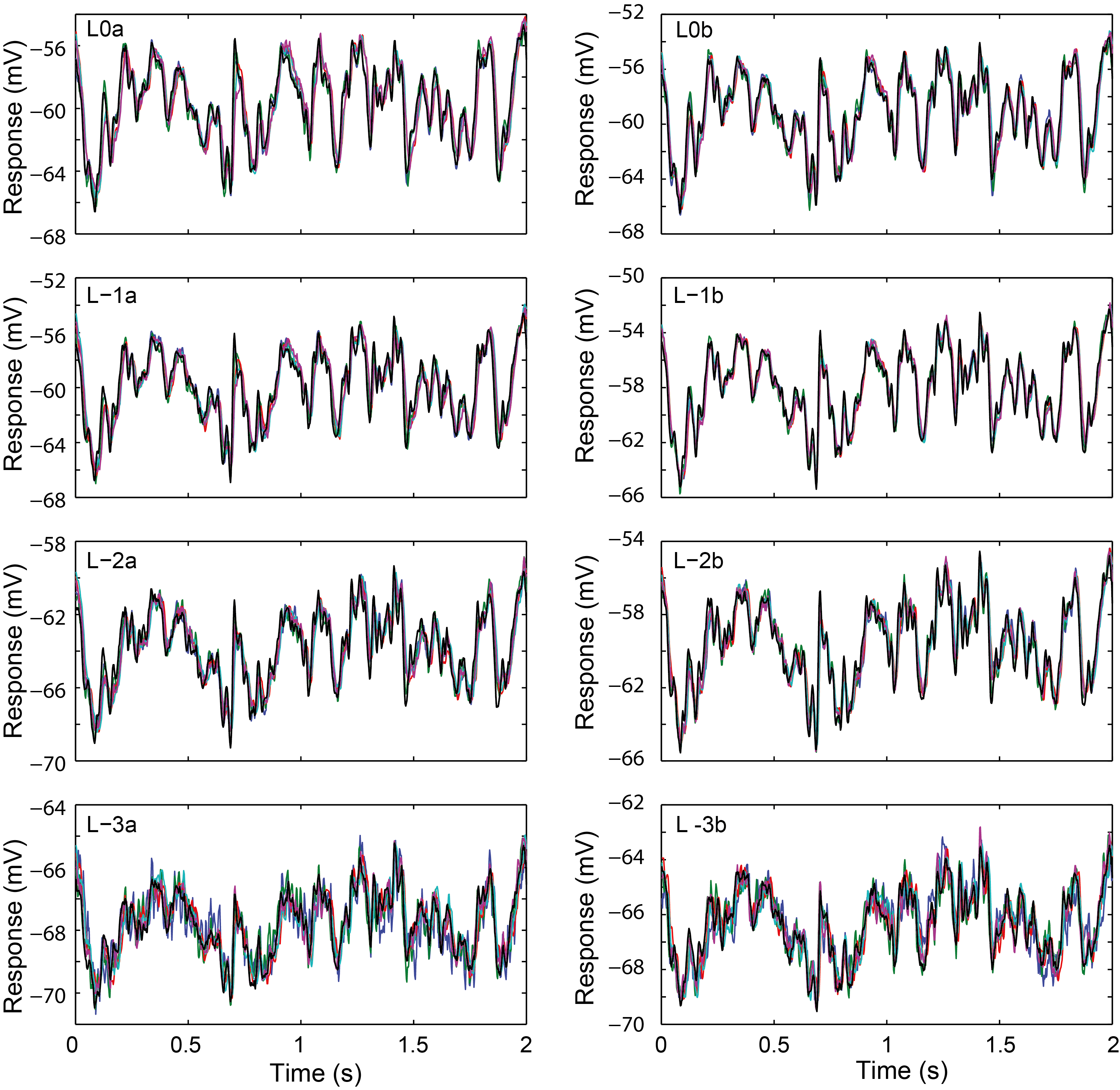

Supplement: S4 Fig — The average photoreceptor response of the six flies for different mean light intensity levels after normalization to a common response offset and deviation. (TIF) [file pone.0157993.s004.tif]

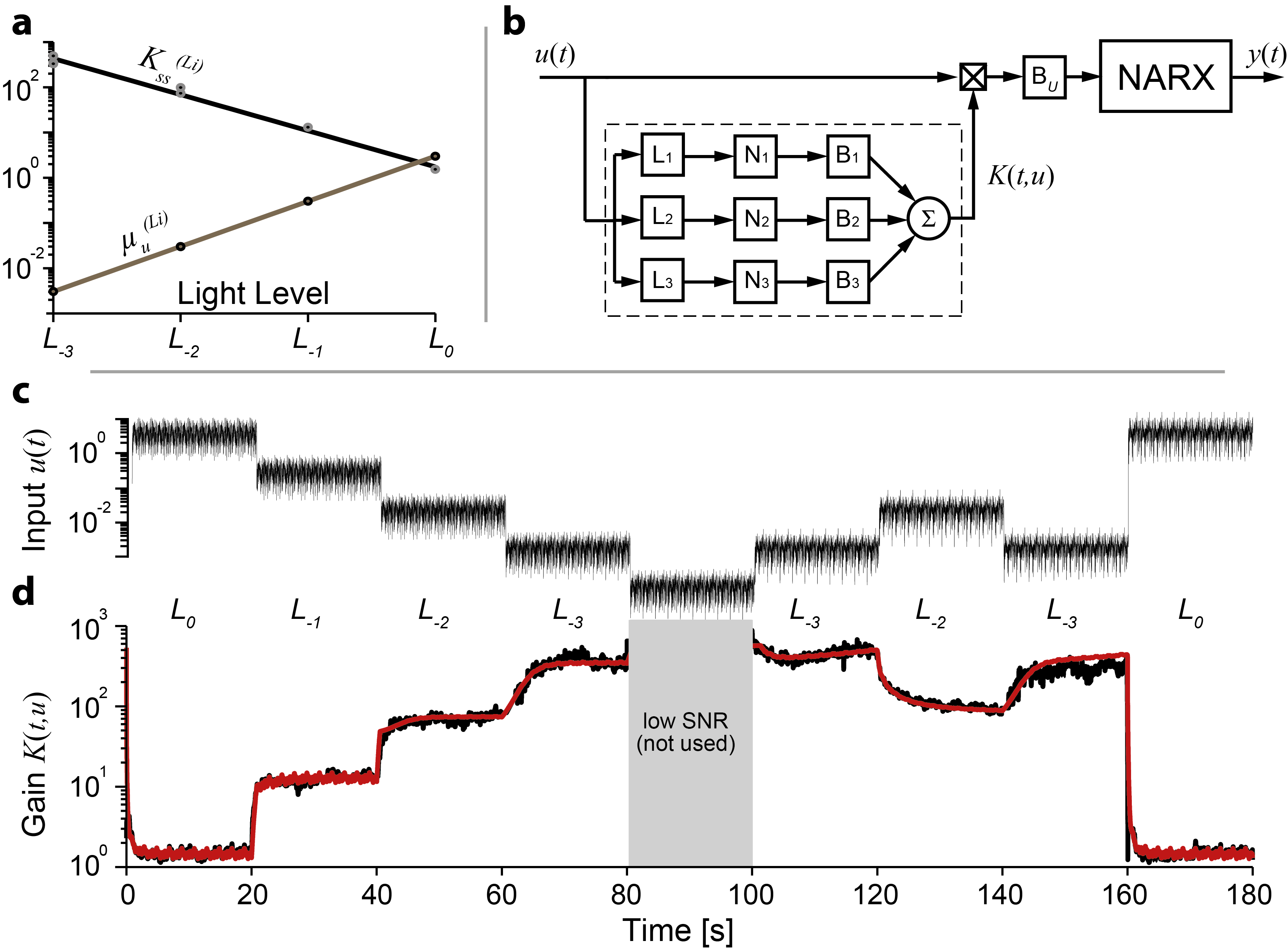

Supplement: S5 Fig — a, Steady state gain (black) vs. mean light intensity (brown). b, Block diagram of the photoreceptor model incorporating the gain control law (dashed-line box). c, Multilevel light contrast stimulus. d, Gain control model predictions (red) superimposed on the estimated gain response (black). (TIF) [file pone.0157993.s005.tif]

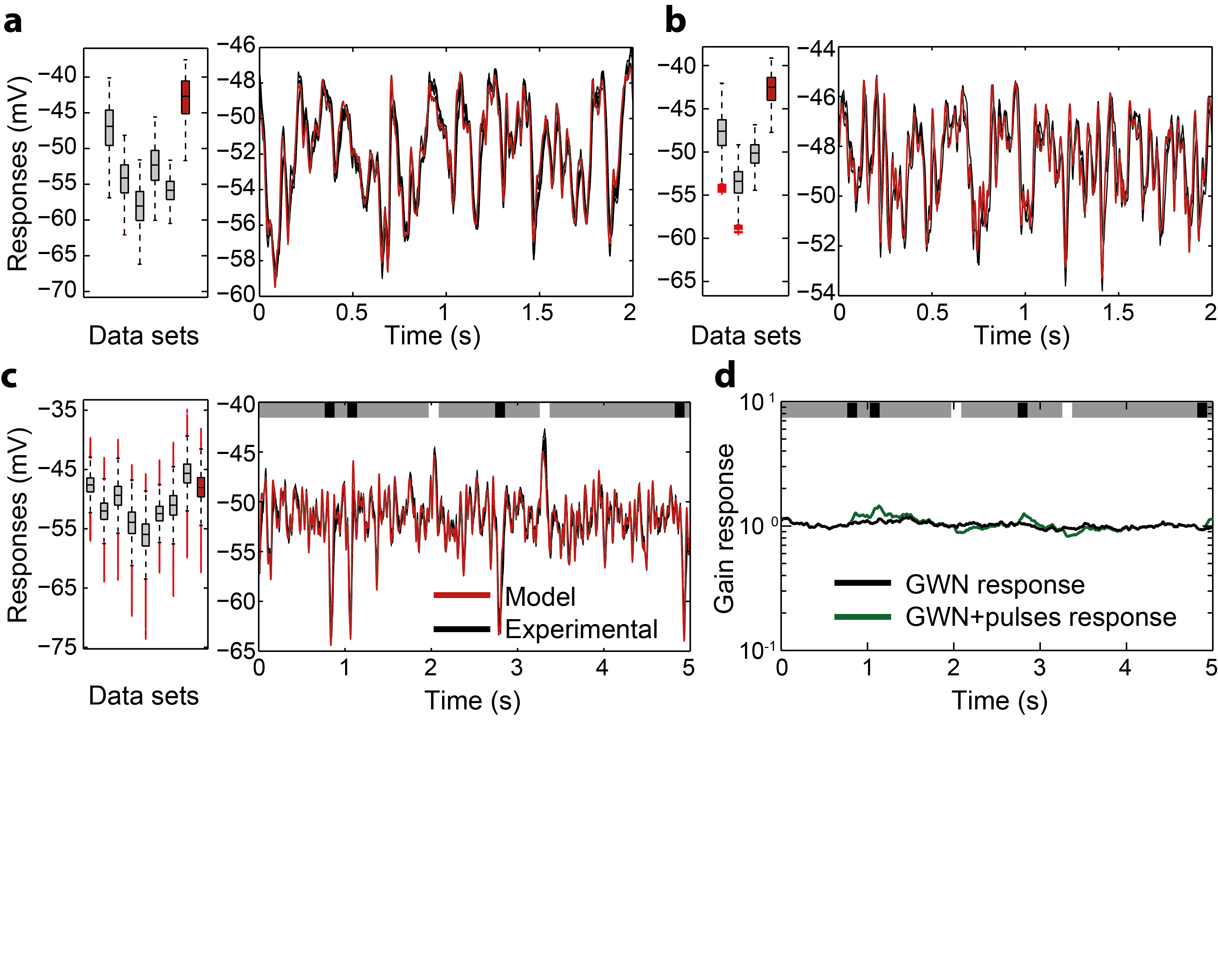

Supplement: S6 Fig — a, Normalized model predictions to a naturalistic stimulus data sequence compared with the average response (n = 8) of 4 cells. b, as in a but for a 100 Hz band limited white noise stimulus sequence for 3 cells. c, as in a but for a stimulus with positive and negative pulses embedded in noise measured in 8 different photoreceptors. Gain response. The box plots are used to show the variations across experimental recordings prior to normalization. (TIF) [file pone.0157993.s006.tif]

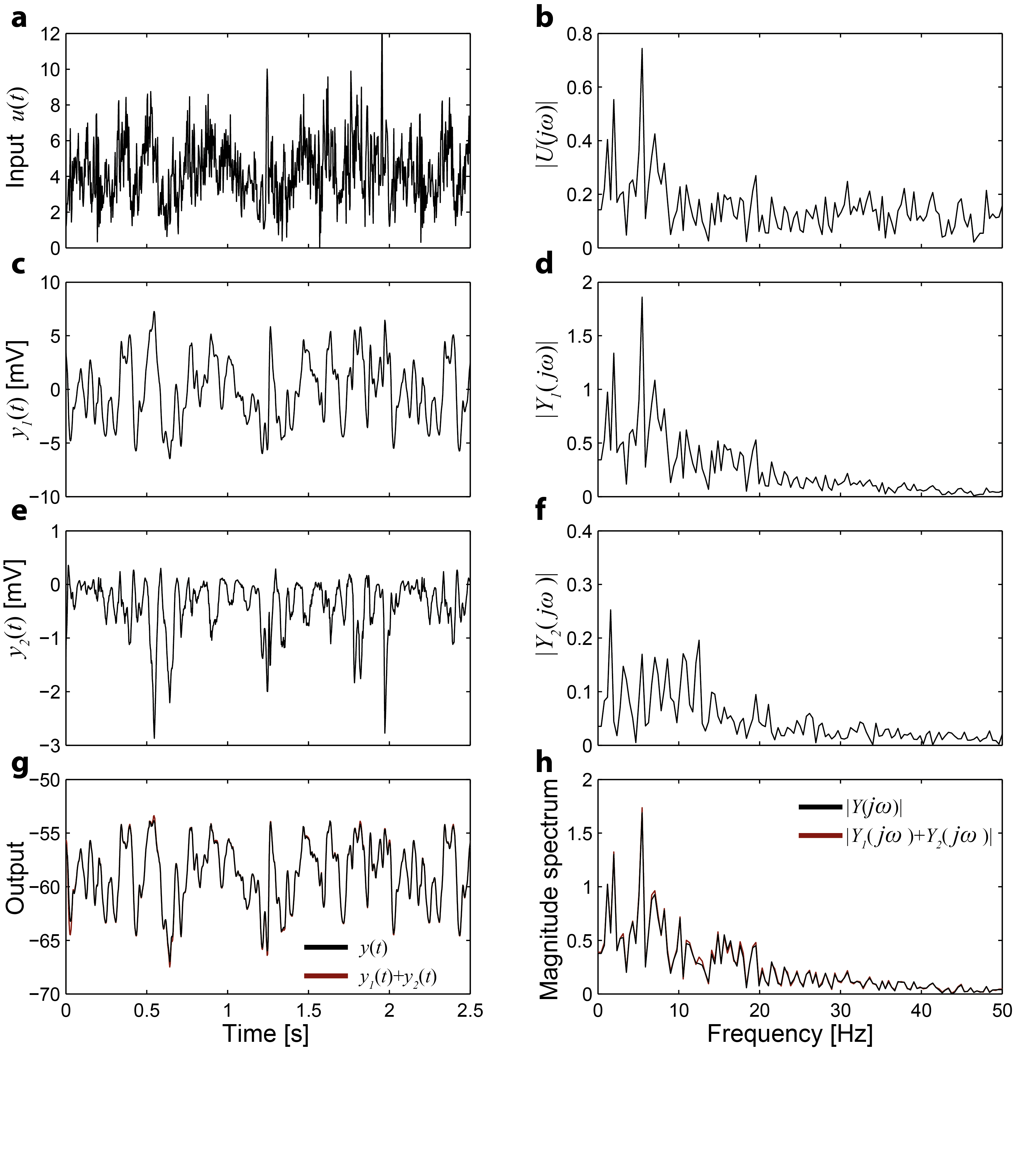

Supplement: S7 Fig — a, Naturalistic input sequence. b, Input spectrum. c, Linear component of the response. d, First-order output frequency response. e, Second-order component of the output. f, Second-order output frequency response. g, Combined first- and second-order time responses (red) match almost perfectly the overall model response (black). h, the combined first- and second-order output frequency response (red) matches the overall output frequency spectrum (black). (TIF) [file pone.0157993.s007.tif]

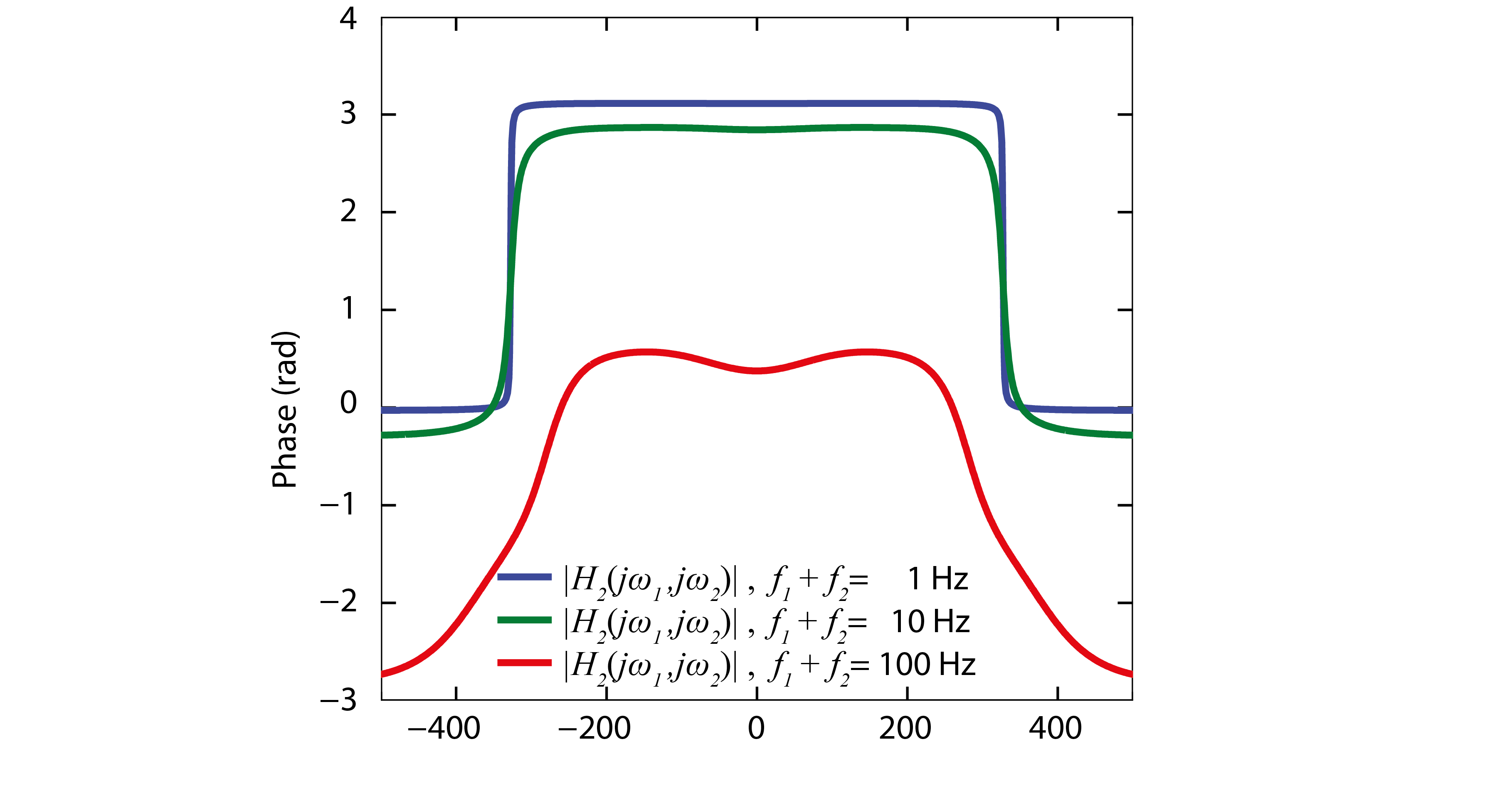

Supplement: S8 Fig — (TIF) [file pone.0157993.s008.tif]

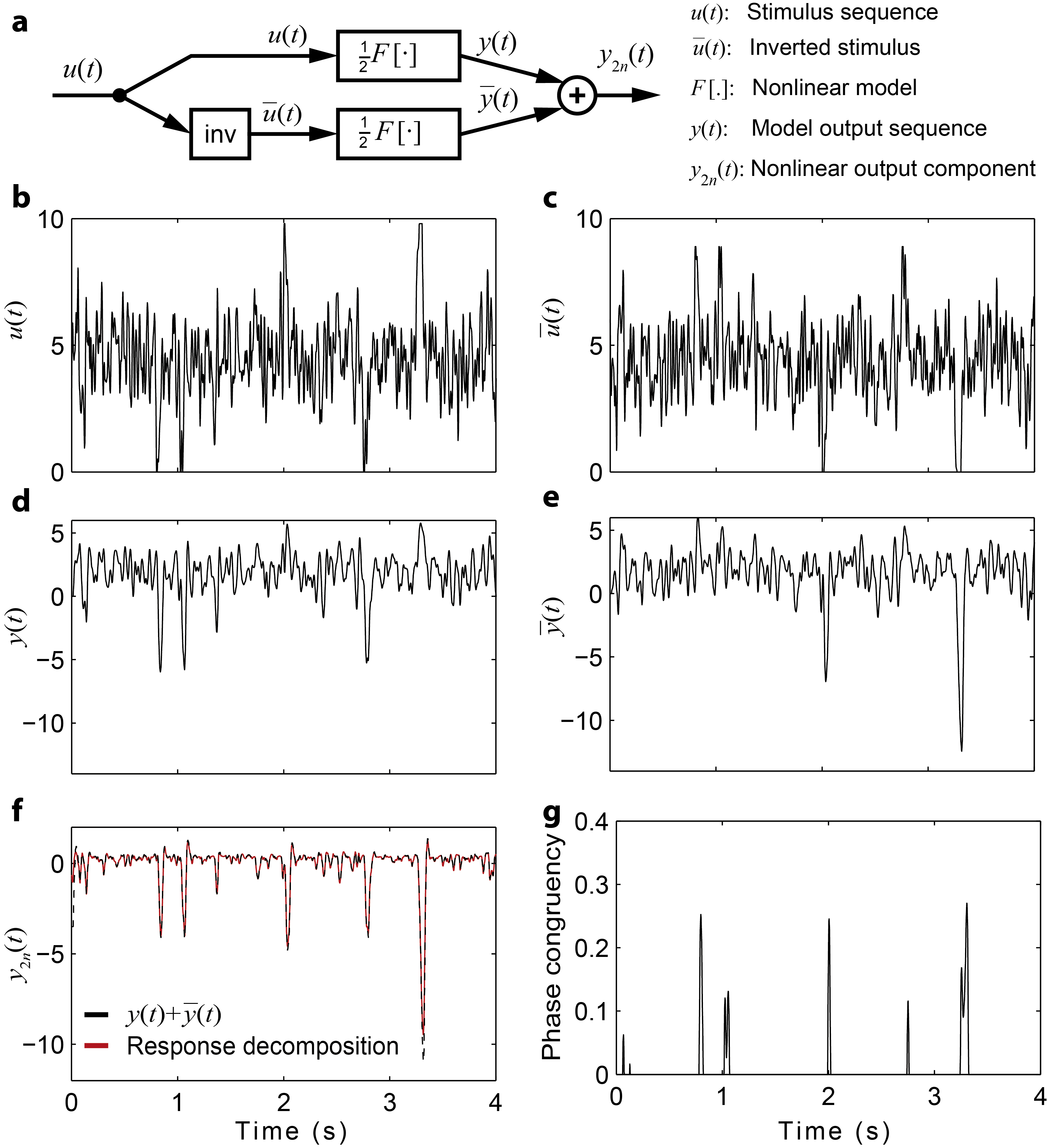

Supplement: S9 Fig — a, Computation of even-order responses. b, Synthetic light stimulus consisting of a sequence of square pulses superimposed on a white noise sequence. c, Inverted version of the stimulus given in b. d and e, Photoreceptor model (mean subtracted) responses to the stimuli given in b and c respectively. f, Even-order response computed by averaging the model predictions shown in d and e, (red) and model predicted nonlinear response (black). g, Local phase congruency measure computed for the synthetic stimulus. (TIF) [file pone.0157993.s009.tif]

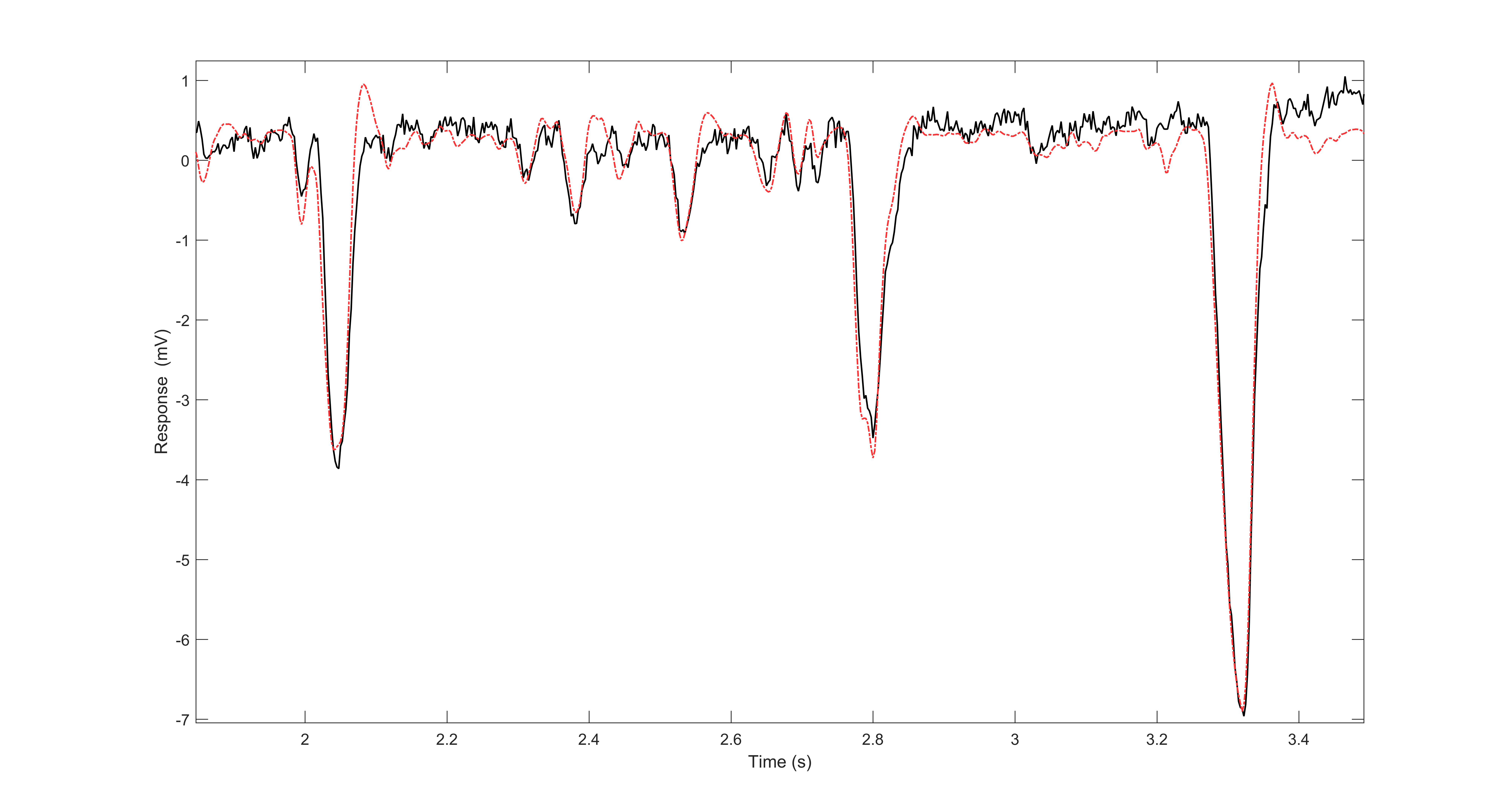

Supplement: S10 Fig — (TIF) [file pone.0157993.s010.tif]

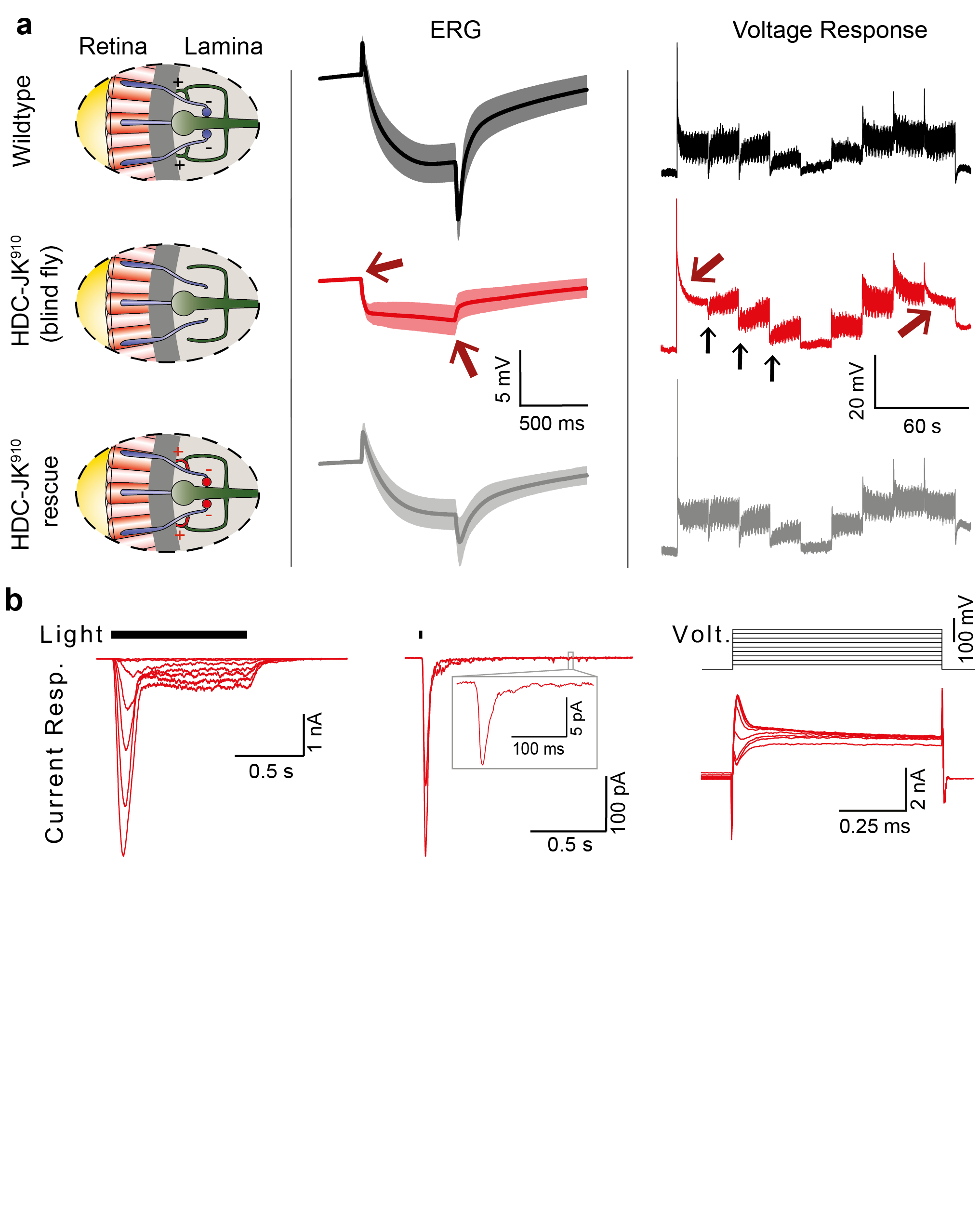

Supplement: S11 Fig — a, ERG and voltage responses measured in photoreceptors of wild-type (black line), hdcJK910 mutant (red line) and rescued hdcJK910 mutant (grey line) flies. ERG voltage responses of histamine deficient hdcJK910 mutants lack on-off transients (red arrows, middle panel), demonstrating that synaptic communication between photoreceptors and lamina interneurons is interrupted. The voltage responses (right panel) suggest that synaptic communication increases the range of environmental light intensities to which R1‐R6 photoreceptors can adapt. Arrows highlight the key differences in mutant photoreceptor responses compared to wild-type responses: contrast saturation for bright stimuli and impaired dynamic adaptation. Both wild‐type and the histamine rescued photoreceptors show normal processing of naturalistic contrast pattern stimuli. b, Whole-cell patch-clamp recordings of current responses to 1s prolonged light and flash light stimuli and to 1s Voltage steps in dissociated hdcJK910 photoreceptors. (TIF) [file pone.0157993.s011.tif]

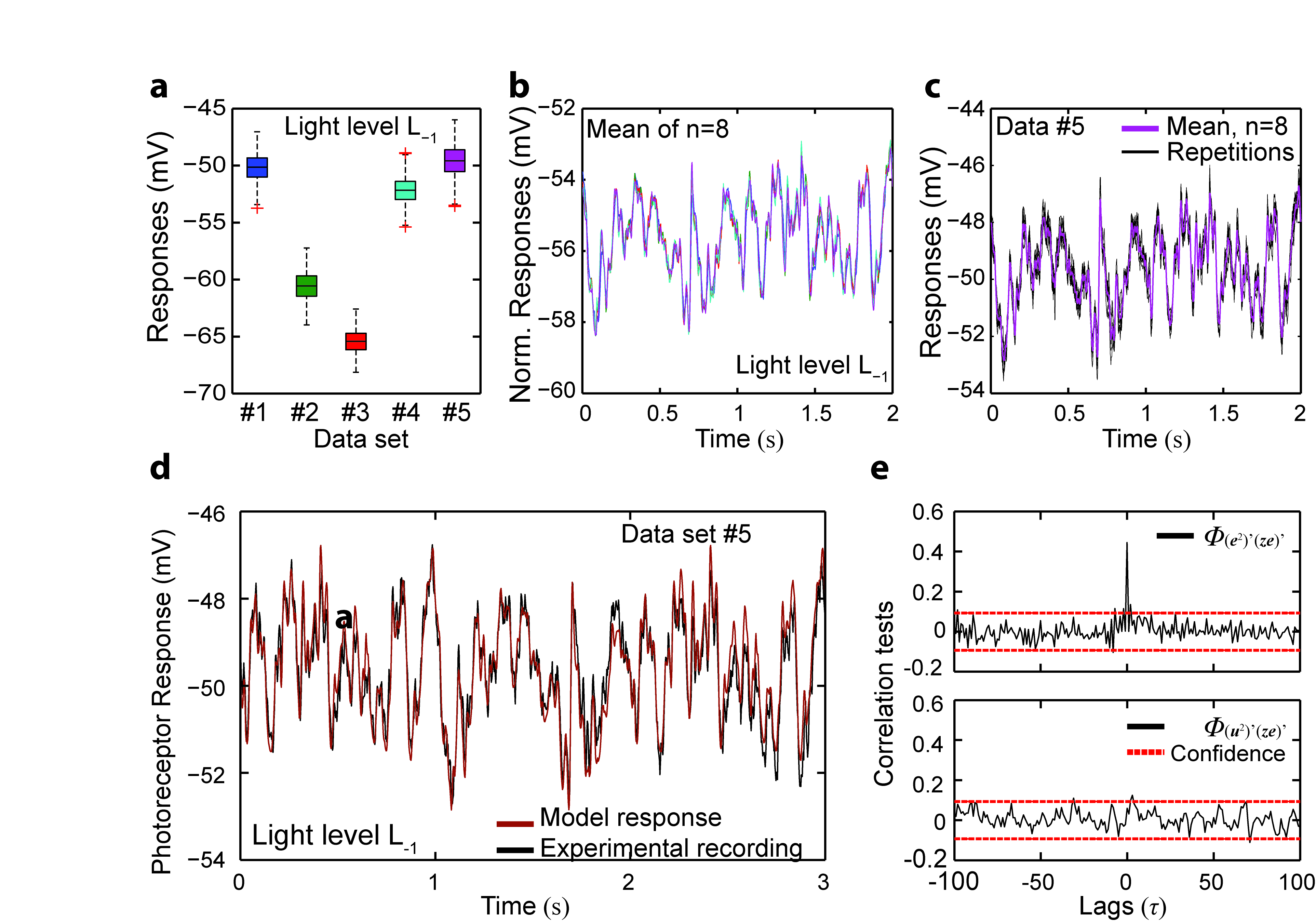

Supplement: S12 Fig — a, Boxplots of response amplitudes during stationary light stimulation at light level L-1 for 5 different flies. b, The mean response based of 8 responses to a single naturalistic stimulus sequence. The mean responses of individual flies are normalized to the mean deviation and amplitude of all flies tested. c, Experimentally measured responses to a repeated stimulus (data set #5) which were used to infer a photoreceptor model. d, Prediction performance of the hdcJK910 photoreceptor model. e, Correlation tests. (TIF) [file pone.0157993.s012.tif]
