# Supplementary material for: Fly Photoreceptors Encode Phase Congruency
Source: PLoS One. 2016 Jun 23;11(6):e0157993. doi: 10.1371/journal.pone.0157993 (PMC4919002; doi:10.1371/journal.pone.0157993)
Supplement: S1 File — (PDF) [file pone.0157993.s013.pdf]

Supplementary Methods

Experimental Data

The stimulus light pattern was specifically designed to elicit a photoreceptors dynamics during both stationary and transient regimes. Here, stationary regimes are characterized by maintaining a constant mean light intensity μ_u and contrast $C_u = \sigma_u / \mu_u$, where σ_u is the signal's standard deviation. Transient regimes are defined to be the time intervals in which the photoreceptor adapts between two different mean light levels $L_i \to L_j$ that have the same contrast.

The light pattern was selected as a naturalistic time-series of intensities (NTSI), chosen from the Van Hateren natural stimuli collection[1]. It was originally collected in a woodland area by moving a highly sensitive light detector along a motion path similar to the trajectory of a flying fly[1]. By replaying the light stimulus to the fly, as described in our experimental procedures a photoreceptor observes to a great extend the same light pattern as if the fly would move through the natural scene. We chose a 2 s representative NTSI interval with a logarithmic power spectrum $S(f)$ that drops with the logarithmic frequency f in the form $S(f) = f^{-1}$ (Figure b in S2 Fig).

To ensure that the photoreceptor response is stationary we synthesized a 20s temporal pattern with constant mean by repeating the selected NTSI interval 10 times. In order to elicit an adaptive transient response, 20s stimuli sequences with mean light intensities, ranging from L_0 (very bright) to L_{-4} (very dim), were concatenated, resulting in a single temporal stimulus sequence that covers the entire operating range of the photoreceptor. The stimuli used are illustrated in S2 Fig.

The mean light intensity changes by a factor of 10 between levels such that $L_0 = 10L_{-1} = 100L_{-2}\ldots$. The light intensity range was deliberately selected to reflect what a fly is normally exposed in nature, such that the brightest stimuli almost saturates the cell, whereas the dimmest stimuli can barely be detected. Shorter adaptation periods did not indicate a change in the adaptation time constants (Figure d in S2 Fig).

To compare data gathered in different experiments and from different flies, the data sets were normalized to a common offset and standard deviation. Data samples for the first 4s after each light level change, which include the transient photoreceptor response (Figure c in S2 Fig), were used to infer the gain control model. The input/output samples for the remaining 16 s were used to estimate a stimulus-invariant model structure. To eliminate the slow adaptive trend of the response for the 16 s data sets were removed by fitting low order polynomials $q(\mathbf{a}_l, t) = \sum_{i=0}^{m} a_{i,l} t^i$, $t = [t_{0,l} + 4, t_{0,l} + 20]$, where $t_{0,l}$, l=1,..,9 are the starting times for each 20s segments of data, m is the polynomial order ($m \leq 7$) and $\mathbf{a}_l = [a_{0,l}, .., a_{m,l}]$ is the coefficient vector to be estimated. An example data set is shown in Figure a in S3 Fig. It is easy to see that the noise in the neural response increases significantly with decreasing light intensity levels. Due to the low signal to noise ratio at the lowest light level L_{-4}, which is 10000 times dimmer than the brightest, these responses were not used in our analysis. The normalized photoreceptor responses to the same NTSI stimulus, recorded in six different flies (S4 Fig), indicate that photoreceptor responses to NTSI data are very similar in different animals.

Identification of a stimulus invariant model structure

The model described in this study was developed using a well-established nonlinear system identification methodology based on the NARMAX model[2,3],

$$y(t) = F[y(t-1),...,y(t-n_y),u(t-1),...,u(t-n_u),e(t-1),...,e(t-n_e)] + e(t)\,, \qquad (0.0)$$

where $y(t)$, $u(t)$ and $e(t)$ are the outputs, inputs and noise variables, respectively; $F[\cdot]$ is an unknown nonlinear function and n_y , n_u and n_e are the maximum output, input and noise lags. The noise variable $e(t)$ accounts for unobserved random perturbations, which are assumed to be zero mean, independent and identical distributed.

In this case, the output $y(t)$ is the intracellular voltage response and the input $u(t)$ is the measured intensity of the light stimulus. The unknown noise signal incorporates measurement as well as noise introduced by the molecular phototransduction mechanisms. Polynomial NARMAX models that can be used to derive analytically GFRFs were estimated initially for each light level. No assumption was made about the model structure or parameters. Model term selection and parameter estimation was performed using an orthogonal forward regression algorithm (OFR) as described in Chen *et al* (1989)[3]. The OFR algorithm selects model terms in a stepwise manner, starting from a large set of candidate polynomial terms. At each step the algorithm selects, from the candidate set, the term which contributes the most to the reduction of the error. The selection process terminates when the estimated model minimizes an information theoretic criteria[4].

The noise model was estimated iteratively, using a prediction error algorithm. Six iterations were carried out to update the noise terms and their coefficients for the noise sequence to converge[3].

Residual analysis was carried out to ensure that the estimated model captured all the underlying dynamics which means that the residuals are uncorrelated with past and inputs and outputs[5].

To avoid overfitting, we implemented a *K*-fold cross-validation procedure (Fig. S5). For each of the 8 stationary stimulus repetitions at each light level only N_e = 600 data samples are taken for the estimation of NARMAX candidate models.

The estimated models were validated on the remaining stationary data of the same light level (N_v =5800 samples).

The maximum number of input and output lags to initialize the regression model was heuristically selected. Best results in terms of prediction performance and model size could be found for $n_y = n_u = 7$. The maximum number of error lags was selected based on a model's performance in the correlation tests. The modelling procedure was repeated for polynomials of increasing order l =1,2,3. While linear models (l =1) failed to predict response peaks, models of dimension (l =3) easily led to over-parameterization and did not perform better than quadratic models (l =2). Thus, only quadratic models were considered.

The models individually identified for each light level contained almost the same set of terms, which varied by merely one or maximal two lags. Consequently, in the second stage, the model structure identified for the brightest level of light L_0 (highest SNR), was used to fit photoreceptor responses for the remaining light levels. The new models, sharing a common model structure, performed equally well, as those estimated in the first step, over both estimation and validation data sets (S6 Fig).

Variable gain model

The response of a wide class of nonlinear dynamical systems in the neighbourhood of a stable equilibrium point can be represented as a Volterra series

$$y(t) = y_0 + \sum_{n=1}^{\infty} y_n(t) \tag{0.0}$$

where y_0 models the *dc* component of the response (i.e. the output is nonzero for zero input excitation) and

$$y_n(t) = \int_{-\infty}^{\infty} \dots \int_{-\infty}^{\infty} h_n(\tau_1, \dots, \tau_n) \prod_{i=1}^{n} u(t-\tau_i) d\tau_i \tag{0.0}$$

where $h_n(\cdot)$ denotes the nth-order Volterra kernel or impulse response. The multidimensional Fourier transform of $h_n(\cdot)$ defines the nth-order Generalized Frequency Response Function $H_n(\cdot)$ such that

$$y_n(t) = \frac{1}{(2\pi)^n} \int_{-\infty}^{\infty} \dots \int_{-\infty}^{\infty} H_n(j\omega_1, \dots, j\omega_n) \prod_{i=1}^{n} U(j\omega_i) \exp(j\omega_i t) d\omega_i \tag{0.0}$$

where $U(j\omega)$ represents the Fourier transform of the input $u(t)$ and $H_0=y_0$.

We used the probing method[6] to compute first (linear) and second-order GFRF functions for the identified NARX models (after discarding the noise model terms) corresponding to each mean light intensity level.

A recursive probing algorithm[6] was used to compute the functional relationship between the identified NARX models and the GFRF's.

As the mean light intensity is reduced (Figure a in S7 Fig) shows that the entire magnitude plot (expressed in dB) of the first- order frequency response functions is shifted up. The second-order log-magnitude functions are shifted by a larger amount which was illustrated by plotting 'slices' through $H_2^{(L_i)}(j\omega_1, j\omega_2)$ along the lines $\omega_1 = \omega_2$ and $\omega_1 = 2\omega_2$ (Figure c in S7 Fig). Importantly, the phase of the frequency response functions does not change (Fig. S7d).

To quantify the changes in the magnitude functions caused by changes of the mean light intensity, we computed the following average shifts of the first- and second-order magnitude functions

$$E_1^{(L_i,L_j)} = \frac{1}{\omega_{max}} \int_{\omega=0}^{\omega_{max}} |H_1^{(L_i)}(\omega)| - |H_1^{(L_j)}(\omega)| \, d\omega \tag{1.5}$$

$$E_2^{(L_i,L_j)} = \frac{1}{\omega_{max}^2} \int_{\omega_1=0}^{\omega_{max}} \int_{\omega_2=0}^{\omega_{max}} |H_2^{(L_i)}(\omega_1, \omega_2))| - |H_2^{(L_j)}(\omega_1, \omega_2))| \, d\omega_1 d\omega_2 . \tag{1.6}$$

The results summarized in figure e in S7 Fig show that

$$E_2^{L_i,L_j} \approx \left(E_1^{L_i,L_j}\right)^2 , \tag{1.7}$$

which suggests that the photoreceptor adaptation to changes in mean light intensity can be modelled by a single input gain parameter such that the photoreceptor response at a given mean light intensity L_k is given by

$$y_{L_k}(t) = F_{L_0}[y_{L_k}(t-1),...,y_{L_k}(t-n_y), K_k u\ (t-1),...,K_k u(t-n_u)] \quad (1.8)$$

Where F_{L_0} is the model (18 parameters) estimated for the brightest stimulus u_0 and $K_0=1$. It follows that

$$\begin{aligned} y^*_{L_k}(t) = K_k \frac{1}{(2\pi)} \int_{-\infty}^{\infty} H_1^{(L_k)}(j\omega) U^*(j\omega) \exp(j\omega t) d\omega + \\ K_k^2 \frac{1}{(2\pi)^2} \int_{-\infty}^{\infty}\int_{-\infty}^{\infty} H_2^{(L_k)}(j\omega_1, \omega_2) U^*(j\omega_1) U^*(j\omega_2) \exp(j\omega_1 t) \exp(j\omega_2 t) d\omega_1 d\omega_2 + ... \end{aligned} \quad (1.9)$$

This explains the log-magnitude shifts shown in Figure e in S7 Fig and thus justifies modelling the photoreceptor adaptation in terms of a single input gain. An output gain, would have shifted equally the log-magnitude plots for the first-and second-order frequency response functions.

We used the model derived earlier for the brightest stimulus as the reference model (as it corresponds to the lowest noise level) to estimate the gain parameters K_{-1}, K_{-2}, K_{-3} for the other light levels $L_{-1}\cdots$ using the Levenberg-Marquardt algorithm. The prediction performance for the single model (S8 Fig) with variable input gain is very good and comparable with that of the previous models with individually estimated parameters or model structures. This unique model with variable gain formed the basis for the subsequent modelling and analysis steps carried out.

Derivation of the Gain control model

To derive a model for the gain control we estimated the gain function $K(t)$ over the entire time course of the experiment. In particular, the 4 s intervals covering the transient photoreceptor response were used to infer the gain adaptation dynamics. This inverse problem is formulated as a nonlinear optimization problem where the cost function is given by

$$\min_{K(t)\in L^2([t_0,t_N])} \sum_{t=t_0}^{t_N} \left| z(t) - F_{L_0}[y(t-1),...,y(t-n_y), K(t)u(t-1),...,K(t)u(t-n_u)] \right|^2, \quad (1.10)$$

where $z(t)$ denotes the measured output.

Solving this infinite-dimensional inverse problem involves approximating the unknown function $K(t)$ on a finite-dimensional subspace $V^n \subset L^2([t_0,t_1])$, spanned by a set of basis functions $\{\varphi_{n,j}(t)\}_{j=1}^T$, by

$$K_n(t) = \sum_j k_j \varphi_{n,j}(t) \quad (1.11)$$

In this case, $\varphi_{n,j}(t) = \sqrt{2^n}\varphi(2^n t - j)$, where $\varphi(t)$ is the 3rd order Battle-Lemarié scaling function[7,8] (Fig. S9b), form an orthonormal basis.

The gain estimation problem reduces to that of estimating k_j which minimise

$$\min_{k_j}\sum_{t=t_0}^{t_N}\left|z(t)-F_{L_0}[y(t-1),...,,u(t-1)\sum_j k_j\varphi_j^{(n)}(t),...,u(t-n_u)\sum_j k_j\varphi_j^{(n)}(t)]\right|^2 \quad (1.12)$$

The optimisation problem (1.12) was solved using the Levenberg-Marquardt algorithm[9]. The approach was validated through numerical simulations using noisy data. The estimated gain response, its initialization and corresponding model predictions are shown in S5 Fig.

The gain control system was modelled as a parallel cascade of three Linear-Nonlinear models. The linear block (*L*), models the dynamics of adaptation

$$\kappa_i \frac{du_i(t)}{dt}+u_i(t)=u(t),\ i=1,2,3\ , \quad (1.13)$$

where the time constant κ_i reflects the fact that photoreceptors have different adaptation mechanisms, which are active over different light intensity ranges and have different characteristic timescales.

We found that the gain control model incorporating three adaptation timescales described very well the dynamics of the photoreceptor gain. Specifically, the three time constants in our model reflect fast adaptation processes (< 1s), consistent with fast calcium dynamics in the photoreceptor's rhabdomere[10], adaption occurring over an intermediate timescale (2-5s), consistent with the pupil mechanism[11], and slow adaptation dynamics (> 10s), which characterize slower processes such as those involving the translocation of signalling proteins[12].

The nonlinear block (*N*) describes the relationship (Figure a in S10 Fig) between the static gain, defined as the mean of $K_n(t)$ during last 4s before a light level changed, and light intensity as

$$K_{ss,i}(\mu_u)=\gamma_i\cdot(\mu_u)^{\nu_i} \quad (1.14)$$

where γ_i is a proportionality factor and $\nu<0$.

Combining the static gain response (1.14) with (1.13) gives

$$\overline{K}_i(t,u(t))=\gamma_i\left(\overline{K}_{0,i}e^{-t/\kappa_i}+\frac{1}{\kappa}\int_0^t e^{-(t-\tau)/\kappa_i}u(\tau)d\tau\right)^{\nu}. \quad (1.15)$$

Since the gain cannot increase above the physiological limitations of the system, a saturation nonlinearity (*B*) is introduced such that

$$K_i=\rho_i+\frac{(\overline{K}_i-\rho_i)}{1+e^{(\overline{K}_i-\rho_i)}}, \quad (1.16)$$

where ρ_i is a parameter which determines the gain saturation limits for each adaptation process.

The gain control law is given by the superposition of three gains

$$K(t,u) = \sum_{i=1}^{3} K_i(t,u) \tag{1.17}$$

The parameter set $M = \{\kappa_i, \nu_i, \gamma_i, \rho_i\}_{i=1}^{3}$ was determined by solving the optimization problem

$$\min_{M} \sum_{t=t_0}^{t_N} \left| z(t) - F_{L_0}[y(t-1),...,y(t-n_y), K(t,u)u(t-1),...,K(t,u)u(t-n_u)] \right|^2 . \tag{1.18}$$

using the Levenberg-Marquardt algorithm[9]. An additional saturation block B_u was used to impose an upper limit on the input signal applied to the NARMAX model. The limit reflects the environmental range of stimuli for which the model was originally estimated. The total number of estimated parameters for the gain control law, including an additional parameter ρ_u corresponding to the input saturation, is 13. The input gain predicted by the estimated gain control model matches very well the estimated gain function. (S5 Fig)

The combined photoreceptor model consisting of the NARX model $F_{L_0}[\cdot]$ and gain control, takes the form

$$y(t) = F_{L_0}[y(t-1),...,y(t-n_y), u_K(t),...,u_K(t-n_u)] \tag{1.19}$$

with

$$u_K(t) = \rho_u + \frac{(K(t)u(t) - \rho_u)}{1 + e^{(K(t)u(t) - \rho_u)}} \tag{1.20}$$

is the upper-bounded, gain-adjusted stimulus.

Model Validation

The photoreceptor model was derived based on a single representative data set of responses to a multi-level naturalistic light contrast pattern. To assess to what extent this is a representative model for *Drosophila* R1-6 photoreceptors; we compared the model responses to a variety of stimuli types with electrophysiological recordings from photoreceptors of a number of different flies.

Recordings from *Drosophila* photoreceptors vary both in their response amplitude and the resting potential. These variations are normal and induced by the difficult experimental procedures and natural variations of photoreceptors. To compare responses of different flies and the model predictions, the responses measured in different flies were normalized to a common mean μ_y and standard deviation σ_y. The normalized response is calculated as

$$y_{norm}(t) = \frac{\sigma_y}{\sigma_{y_{exp}}} \left(y(t) - \mu_{y_{exp}} \right) + \mu_y . \tag{1.21}$$

where

$$\mu_y = \frac{1}{n_{exp}} \sum_{\exp=1}^{n_{exp}} \mu_{y_{\exp}} , \quad \sigma_y = \frac{1}{n_{exp}} \sum_{\exp=1}^{n_{exp}} \sigma_{y_{\exp}} , \tag{1.22}$$

$$\mu_{y_{exp}} = \frac{1}{N_y}\sum_{i=1}^{N_y} y(t_i) \text{ and } , \sigma_{y_{exp}} = \sqrt{\frac{1}{N_y}\sum_{i=1}^{N_y}(y(t_i)-\mu_{y_{exp}})^2} , \quad (1.23)$$

where n_{exp} is the number of data sets available and N_y is the number of samples.
The original model was used in all simulations without any re-adjustments to its parameters. The model predictions were compared in each case with the normalized responses.
The stimuli patterns used in experiments and simulation included a naturalistic light contrast pattern, a bandlimited normal distributed noise sequence and a sequence of pulses embedded in noise. The naturalistic sequence was taken from the van Hateren naturalistic stimulus library[1]. The white noise sequence (generated in Matlab (R2009b)) was low-pass filtered using a Butterworth IIR filter with a cut off frequency of 100Hz chosen to match photoreceptor bandwidth.
The third stimulus is similar to that shown in Figure 3a. This stimulus was only tested at light level L_0, whereas the naturalistic and noise stimuli were also tested on light levels L_{-1} to L_{-3} (not shown here). Each stimulus was repeated 10 times for each fly, of which the later 8 repetitions were averaged to increase the signal to noise ratio (S6 Fig). For all tested data stimulus sequences after normalization, the model predicted responses resembled remarkably well the averaged responses of photoreceptors from different flies. The same is true for the lower light levels which are not explicitly shown here.

The gain response for the pulse signal is only marginally different compared to the gain response elicited by the same signal with the pulses removed. This emphasizes the fact that the edge detection mechanisms are not related to gain dynamics; this was also verified by forcing the gain to be constant

Response Decomposition using the Output Frequency Response Functions

The output frequency responses (OFR) were computed based on the GFRF's $H_1(j\omega)$ and $H_2(j\omega_1, j\omega_2)$.
The output spectrum of the photoreceptor model subjected to an arbitrary input $u(t)$ with constant gain is given by

$$Y(j\omega) = \sum_{n=1}^{N} Y_n(j\omega) \quad (1.24)$$

where

$$Y_n(j\omega) = \frac{1/n^{1/2}}{(2\pi)^n} \int_{\omega=\omega_1+\ldots+\omega_n} H_n(j\omega_1,\ldots,j\omega_n)\prod_{i=1}^{n} U(j\omega_i)d\sigma_\omega$$

describes the input contribution to the output spectrum at the frequency ω, through n-th order nonlinear interactions. For the stimuli sequences considered in the paper we computed the output spectra $Y_1(j\omega), Y_2(j\omega)$ and then subsequently $y_1(t)$ and $y_2(t)$ shown in S7 Fig, which combined were found to provide very good approximation to the overall response. We based OFR computation on Lang et al, 2007[13].

In practice, the even-order responses can be computed directly from the experimental recordings by stimulating the photoreceptor alternatively with two input signals $u(t)$ and $\bar{u}(t)$

$$\bar{u}(t) = -u(t) + 2\mu_u, \tag{1.25}$$

where μ_u is the mean of $u(t)$, such that

$$\angle\bar{U}(j\omega) = \angle U(j\omega) + 180^0 \tag{1.26}$$

For even ($2l$) and odd ($2l+1$) output spectra it follows that

$$\begin{aligned} Y_{2l}^{U(j\omega)}(j\omega) &= Y_{2l}^{\bar{U}(j\omega)}(j\omega) \\ Y_{2l+1}^{U(j\omega)}(j\omega) &= -Y_{2l+1}^{\bar{U}(j\omega)}(j\omega) \end{aligned} \tag{1.27}$$

Therefore, averaging photoreceptor responses to the two inputs will cancel the odd-order responses, including the linear response that makes the largest contribution to the overall response. The resulting signal is a superposition of all even-order responses

$$y_{2l}^{*}(t,K) = \sum_{l=1} y_{2l}^{*}(t,K) \cong y_{2}^{*}(t,K)$$

which in our case matches very closely the second-order response because the higher-order contributions are negligible.

Modelling and Analysis of Synaptically Isolated Photoreceptors

Fly photoreceptors use the neurotransmitter histamine to communicate visual information to interneurons[16]. In histamine deficient mutants, the lamina interneurons fail to receive and transmit visual information and their feedback synapses can no longer modulate the photoreceptor output[17]. Here, *hdc*$^{\mathrm{JK910}}$ fly mutants[17], were used to test if the lamina network contributes towards local and global phase processing in photoreceptors. For this purpose modeling and analysis procedures used above for testing wild type photoreceptors were repeated on *hdc*$^{\mathrm{JK910}}$ fly mutants and despite the lacking network the cells elicited similar processing properties.

Mutations can have unexpected side-effects, or mutant flies may carry hidden secondary mutations, tampering the electrophysiological results. To assure that the phototransduction cascade is unaffected by the *hdc* mutation, whole cell patch-clamp recordings were performed on dissociated mutant fly photoreceptors as described in reference[18]. Voltage clamp recordings from mutant photoreceptors stimulated by prolonged and brief light flashes or to voltage step signals (Figure b in S10 Fig.) elicit very similar characteristic current responses as known from wild-type photoreceptors[18]. Mutant fly currents differ only by a small reduction of quantum efficiency and slightly lower capacitance values. While mutant current responses elicited an unusual slow tail ($\tau \approx 100\,ms$), their quantum bump dynamics provided no significant differences to wild type photoreceptors. The minor differences between wild type and *hdc*$^{\mathrm{JK910}}$ fly mutant current responses of isolated cells are a possible reason for slight differences between the GFRFs derived for wild type (main article Fig. 2b) and mutant photoreceptor dynamics (main article Fig. 6b).

To test that there is indeed no connectivity between photoreceptors and lamina neurons, electroretinogram (ERG) recordings and behavioural experiments were

performed. ERG's from histamine mutant flies to 700ms light steps showed no on or off transients, which are characteristic for LMC cell responses and apparent in wild type photoreceptors (Figure a in S10 Fig). In behavioural experiments performed according to reference[19], mutant flies showed no behavioural response to moving grading stimuli in the flight simulator. Rescuing the mutant flies histamine deficiency by feeding them histamine 2 days before the experiments according to reference[17] recovered both the ERG transients (S10 Fig) and the behavioural responses.
The fact that photoreceptor responses to naturalistic and white noise stimuli in histamine-fed mutant flies' are very close to wild type responses indicate that the missing synaptic connectivity is the major difference between the two fly phenotypes. Therefore, histamine mutant are an appropriate choice to test how the lamina network contributes towards photoreceptor processing.

Recordings of Intracellular Voltage Responses during Multilevel Naturalistic Light Stimulation
For a direct comparison of hdc^{JK910}fly mutant to wild type photoreceptor processing, the same recordings to multilevel naturalistic light pattern (Fig. S2) were repeated on mutant flies. At the brightest light level hdc^{JK910} photoreceptors saturate (S10 Fig). At all other light levels the response amplitude is slightly reduced and the resting potential is a bit higher than for wild type recordings. In total, 5 reliable recordings to the multilevel stimulus paradigm were obtained. Similar to wild type recordings, the response variance and offset varied significantly between different animals. This difference between recordings can be either due to natural variations in the cell dynamics, or is down to experimental difficulties. As with the wild type flies, the normalized responses from different flies to the same stimuli, are highly correlated.

The hdc mutant photoreceptor model
Mutant photoreceptors saturate at the brightest light level L_0 (S10 Fig) and the highest signal to noise ratio of responses is achieved at light level L_{-1}. This suggests that the retinal network contributes to extend the range of light intensities to which the photoreceptor can adapt. Due to saturation, the responses for the brightest (L_0) stimulus sequence have a very low signal to noise ratio. For the other, lower intensity stimulus sequences, the SNR is also significantly lower.
Recordings obtained at this light level were used to estimate the model. We used $N_e = 600$ data samples for estimation and $N_v = 5800$ for model validation. We found that the model structure derived for the wild type photoreceptor was adequate and so only the model parameters and the noise model had to be re-estimated. The hdc^{JK910} mutant photoreceptor model was validated as described in the wild type case. The performance of the model is similar to that achieved by the wild type model (S11 Fig). In particular, the normalized model prediction error, computed over the entire data set (*NMSE*=0.092) is comparable to that obtained for wild-type photoreceptors at the same light level. Due to the limited range, in which the photoreceptors of mutant flies can operate, we did not attempt to derive a gain control model. Accordingly, all the simulations carried used stimuli scaled to light level $L_{-1}=L_0/10$.

Supplementary References
1 van Hateren, J. H. Processing of natural time series of intensities by the visual system of the blowfly. *Vision Research* **37**, 3407-3416 (1997).

2 Leontaritis, I. J. & Billings, S. A. Input-Output Parametric Models for Non-Linear Systems Part I: Deterministic Non-Linear Systems. *International Journal of Control* **41**, 303-344 (1985).
3 Chen, S., Billings, S. A. & Luo, W. Orthogonal least-squares methods and their application to non-linear system-identification. *International Journal of Control* **50**, 1873-1896 (1989).
4 Akaike, H. Statistical predictor identification. *Annals of the Institute of Statistical Mathematics* **22**, 203-217, doi:10.1007/bf02506337 (1970).
5 Billings, S. A. & Zhu, Q. M. Nonlinear model validation using correlation tests. *International Journal of Control* **60(6)**, 1107-1120 (1994).
6 Peyton Jones, J. C. & Billings, S. A. Recursive algorithm for computing the frequency response of a class of non-linear difference equation models. *Internationa Journal of Control* **50**, 1925--1940 (1989).
7 Battle, G. A block spin construction of ondelettes. Part I: Lemarié functions. *Communications in Mathematical Physics* **110**, 601-615, doi:10.1007/bf01205550 (1987).
8 Lemarié, P. G. Ondelettes à localisation exponentielle. *Journal de Mathematiques Pures et Appliquees* **67** 227-236 (1988).
9 Seber, G. A. F. & Wild, C. J. *Computational methods for nonlinear least squares*. (John Wiley & Sons, Inc., 2005).
10 Hardie, R. C. *et al.* Protein kinase C is required for light adaptation in Drosophila photoreceptors. *Nature* **363**, 634-637 (1993).
11 Kirschfeld, K. & Franceschini, N. Ein mechanismus zur steuerung des lichtflusses in den rhabdomeren des komplexauges von *Musca*. *Biological Cybernetics* **6**, 13-22, doi:10.1007/bf00288624 (1969).
12 Frechter, S. & Minke, B. Light-regulated translocation of signaling proteins in *Drosophila* photoreceptors. *Journal of Physiology* **99**, 133-139, doi:DOI: 10.1016/j.jphysparis.2005.12.010 (2006).
13 Lang, Z. Q., Billings, S. A., Yue, R. & Li, J. Output frequency response function of nonlinear Volterra systems. *Automatica* **43**, 805-816, doi:10.1016/j.automatica.2006.11.013 (2007).
14 Haykin, S. S. & Van Veen, B. *Signals and systems*. 2nd Edition edn, (Wiley, 2002).
15 Gonzalez-Bellido, P. T., Wardill, T. J. & Juusola, M. Compound eyes and retinal information processing in miniature dipteran species match their specific ecological demands. *Proceedings of the National Academy of Sciences*, doi:10.1073/pnas.1014438108 (2011).
16 Hardie, R. C. A histamine-activated chloride channel involved in neurotransmission at a photoreceptor synapse. *Nature* **339**, 704-706 (1989).
17 Melzig, J., Burg, M., Gruhn, M., Pak, W. L. & Buchner, E. Selective histamine uptake rescues photo- and mechanoreceptor function of histidine decarboxylase-deficient *Drosophila* mutant. *Journal of Neuroscience* **18**, 7160-7166 (1998).
18 Hardie, R. C. Whole-cell recordings of the light-induced current in Drosophila photoreceptors: evidence for feedback by calcium permeating the light sensitive channels. *Proceedings of the Royal Society B: Biological Sciences* **245**, 203-210, doi:10.1098/rspb.1991.0110 (1991).
19 Wardill, T. J. *et al.* Multiple Spectral Inputs Improve Motion Discrimination in the Drosophila Visual System. *Science* **336**, 925-931 (2012).
