# Supplementary material for: Fly Photoreceptors Encode Phase Congruency
Source: PLoS One. 2016 Jun 23;11(6):e0157993. doi: 10.1371/journal.pone.0157993 (PMC4919002; doi:10.1371/journal.pone.0157993)
Supplement: S1 Table — (DOCX) [file pone.0157993.s014.docx]

**S1 Table.** Relative mean square prediction error calculated using the model predicted output and normalized photoreceptor responses, measured in six flies (S4 Fig), to the naturalistic stimuli with different mean light intensity levels.
