# Supplementary material for: Fly Photoreceptors Encode Phase Congruency
Source: PLoS One. 2016 Jun 23;11(6):e0157993. doi: 10.1371/journal.pone.0157993 (PMC4919002; doi:10.1371/journal.pone.0157993)
Supplement: S2 Table — (DOCX) [file pone.0157993.s015.docx]

**S1 Table.** Relative mean square prediction error calculated using the model predicted output and normalized photoreceptor responses, measured in three flies (level L_0_ responses shown in S6 Fig b), to bandlimited (100 Hz) white noise stimuli with different mean intensity levels.
