# Supplementary material for: Fly Photoreceptors Encode Phase Congruency
Source: PLoS One. 2016 Jun 23;11(6):e0157993. doi: 10.1371/journal.pone.0157993 (PMC4919002; doi:10.1371/journal.pone.0157993)
Supplement: S3 Table — (DOCX) [file pone.0157993.s016.docx]

**S3 Table.** Relative mean square prediction error calculated using the model predicted output and normalized photoreceptor responses, measured in eight flies (S6 Fig c), to the GWN+pulses stimulus sequence corresponding to a mean light intensity level L_0_.
